# Osteoarthritis: pathogenic signaling pathways and therapeutic targets

**DOI:** 10.1038/s41392-023-01330-w

**Published:** 2023-02-03

**Authors:** Qing Yao, Xiaohao Wu, Chu Tao, Weiyuan Gong, Mingjue Chen, Minghao Qu, Yiming Zhong, Tailin He, Sheng Chen, Guozhi Xiao

**Affiliations:** 1 Department of Biochemistry, School of Medicine, Shenzhen Key Laboratory of Cell Microenvironment, Guangdong Provincial Key Laboratory of Cell Microenvironment and Disease Research, Southern University of Science and Technology, Shenzhen, 518055 China; 2 Department of Orthopaedics, Union Hospital, Tongji Medical College, Huazhong University of Science and Technology, Wuhan, 430022 China

**Keywords:** Rheumatic diseases, Senescence, Molecular medicine

## Abstract

Osteoarthritis (OA) is a chronic degenerative joint disorder that leads to disability and affects more than 500 million population worldwide. OA was believed to be caused by the wearing and tearing of articular cartilage, but it is now more commonly referred to as a chronic whole-joint disorder that is initiated with biochemical and cellular alterations in the synovial joint tissues, which leads to the histological and structural changes of the joint and ends up with the whole tissue dysfunction. Currently, there is no cure for OA, partly due to a lack of comprehensive understanding of the pathological mechanism of the initiation and progression of the disease. Therefore, a better understanding of pathological signaling pathways and key molecules involved in OA pathogenesis is crucial for therapeutic target design and drug development. In this review, we first summarize the epidemiology of OA, including its prevalence, incidence and burdens, and OA risk factors. We then focus on the roles and regulation of the pathological signaling pathways, such as Wnt/β-catenin, NF-κB, focal adhesion, HIFs, TGFβ/ΒΜP and FGF signaling pathways, and key regulators AMPK, mTOR, and RUNX2 in the onset and development of OA. In addition, the roles of factors associated with OA, including MMPs, ADAMTS/ADAMs, and PRG4, are discussed in detail. Finally, we provide updates on the current clinical therapies and clinical trials of biological treatments and drugs for OA. Research advances in basic knowledge of articular cartilage biology and OA pathogenesis will have a significant impact and translational value in developing OA therapeutic strategies.

## Introduction

**Fig. 1:**
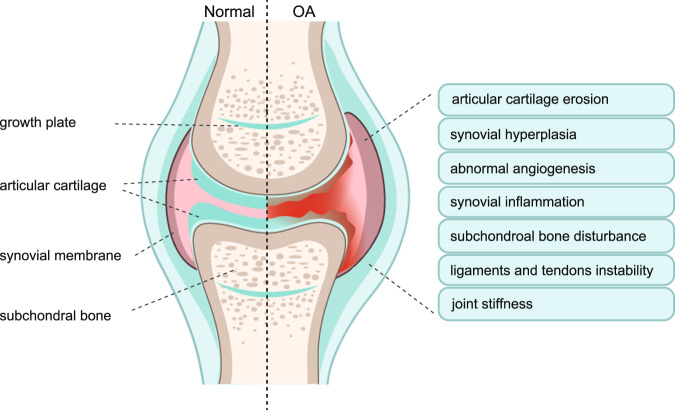
Phenotypes of Osteoarthritis (OA). Clinic evidence shows that the majority of OA patients have a diversity of OA phenotypes, including articular cartilage erosion, synovial hyperplasia, abnormal angiogenesis, synovial inflammation, subchondral bone disturbance, ligaments and tendons instability, and joint stiffness. Left-half side shows the structure of the normal synovial joint. Right-half side showed the possible alterations of synovial joint structure and symptoms in osteoarthritis

Osteoarthritis (OA) is one of the most common types of arthritis and a chronic degenerative and disabling disease characterized by complex disorders of the whole synovial joint,^1^ including structural defects of hyaline articular cartilage, loss of intact subchondral bone, tissue hypertrophy and increasing of vascularity in the synovium, and instability of the tendons and ligaments (Fig. 1). In 2021, >22% of adults older than 40 had knee OA, and it is estimated that over 500 million individuals are currently affected by OA worldwide.^2^ Lacking long-term clinical treatment, OA patients at the end-stage of the disease are ultimately subjected to joint replacement surgery. Joint replacement surgery is growing at a rate of 10% per year globally, and 95% is performed for OA patients.^3^ However, the lifespan of the artificial joint is limited, and the risk of poor outcomes exists. By 2020, OA is globally estimated to be the fourth leading cause of disability, with a huge amount of medical and healthcare costs and indirect costs caused by loss of jobs and early retirement.

There is currently no cure for OA. It has been a long time since clinical treatments of OA focused on improving joint pain symptoms rather than on the decline of the disease progression. In recent years, strategies for OA have been shifted to its early prevention and halt or delay OA progression before massive destruction occurs. Therefore, understanding and identifying potential biomarkers and therapeutic targets at different stages of OA are urgent. Scientists and clinicians have devoted great efforts to defining major signaling pathways and molecules that play essential roles in the initiation and development of OA and could finally be developed as potential therapeutical targets to slow down or limit the damage to synovial joints.

Besides the updated epidemiology of OA, including its prevalence, incidence, burden, and risk factors, we have reviewed our current understanding of the pathogenesis in terms of synovial tissue interactions and cellular biology in OA, as well as pathological signaling pathways and essential molecules of OA. We have summarized the roles and functions of those pathological molecular signaling pathways and key molecules in different components of the synovial joints at different stages of OA and their related clinical relevance. We have finally reviewed current clinical therapies applied to OA patients and updated clinical trials of new drugs and biological treatments for future OA treatment.

## Prevalence

Osteoarthritis (OA) is among the most prevalent diseases globally, which affects multiple joints, including the hip, knee, ankle, hand, and temporal-mandibular joint (TMJ) and other joints.^1,4,5^ The knee, hand, and hip joints are most susceptible to developing OA.^6^ During the past century, the prevalence of OA has grown rapidly in part due to recent increases in lifespan and body weight.^7,8^ According to a large cohort study in the United States, the prevalence of knee OA has increased by 2.1-fold since the 1950s.^9^ It is anticipated that, by 2032, the prevalence of OA will rise from 26.6% to 29.5%.^10^ The prevalence rate of OA can be variable in different studies based on the definitions of OA, e.g., radiographic OA or symptomatic OA. In general, radiographic OA is more prevalent than symptomatic OA.^11,12^ The Global Burden of Disease (GBD) study showed that the global OA prevalence was about 300 million in 2017 and rapidly increased to 530 million in 2019.^13,14^ The prevalence rate of OA has elevated by 9.3% from 1990 to 2017 and by 13.25% from 1990 to 2019.^13,14^ The prevalence standardized by age was estimated at 6,173.38 per 100,000 in 1990 and 6,348.25 per 100,000 in 2019.^14^ In 2019, China was reported to have the largest number of prevalent OA cases (132.81 million), followed by India (62.36 million) and the U.S. (51.87 million).^14^ Moreover, the percentages of OA prevalence in China, India, and the United States have dramatically increased by 156.58%, 165.75%, and 79.63%, respectively, from 1990 to 2019.^14^ The global prevalence of OA in 2019 was significantly higher in females (317.44 million) than in males (210.37 million) and was markedly increased in older people, with a peak level in the 60–64 age group.^14^ A recent study has developed an algorithm to identify patients with hip OA and conducted a retrospective cohort study using this algorithm together with data from the Integrated Primary Care Information database.^15^ The results revealed that the prevalence of hip OA increased from 4.03% in 2008 to 7.34% in 2019.^15^

The prevalence of OA varies in different regions.^14,16–18^ Epidemiological studies from the 2019 GBD study showed that, after standardization by age, sex, and sociodemographic index, the top three regions of OA prevalence in 2019 were East Asia (137.3 million), South Asia (75.6 million), and Western Europe (57.0 million) (Table 1). In contrast, the prevalence of OA was significantly lower in Oceania (0.4 million (95% UI 0.3–0.4 million)), the Caribbean (3.0 million (95% UI 2.7–3.4 million)) and Central Sub-Saharan Africa (3.2 million (95% UI 2.9–3.6 million)) (Table 1). The standardized prevalence of OA was about 10.7% in the United Kingdom and has gradually increased at an annual rate of 1.4%.^19^ In China, the prevalence rates of symptomatic knee OA were estimated at 8.1% (5.7% in men and 10.3% in women) in 2012 and 17% (12.3% in men and 22.2% in women) in 2018 based on a population-based meta-analysis.^20,21^ No marked difference was observed in OA prevalence between the northern and southern areas in China.^20^ However, the prevalence rates of knee OA were much higher in rural areas of China than in urban regions of China.^22^ Another study analyzed the overall prevalence of different types of OA in the middle-aged and elder populations in China and reported that lumbar OA was with the highest prevalence (25.03%), followed by knee OA (21.51%) and cervical OA (20.46%).^23^ Epidemiologists have compared the OA prevalence between China and the U.S. Interestingly, the results showed that both radiographic and symptomatic OAs were more prevalent in women from China than in women from Framingham, Massachusetts.^24^ In contrast, hip and hand OAs were less prevalent in China than in the United States.^25,26^

**Table 1:** Prevalent cases, age-standardized rate and percent for osteoarthritis in 2019 for both sexes, and percentage change of age-standardized rates by Global Burden of Disease regions

	Counts millions (2019)			Percent % (2019)			Age-standardized estimates (2019)			Annual rate of change in age-standardized rates between 1990 and 2019
	Women	Men	Total	Women	Men	Both	Women	Men	Both	
Global	317.4 (350.3–288.5)	210.4 (190.2–233.7)	527.8 (478.7–584.8)	8.46 (7.69–9.34)	5.71 (5.16–6.32)	7.09 (6.43–7.85)	7278.0 (6613.6–8038.6)	5324.0 (4827.0–5885.2)	6348.3 (5776.3–7023.0)	3.9 (2.8–5.1)
Andean Latin America	2.1 (2.3–1.9)	1.4 (1.2–1.5)	3.5 (3.2–3.8)	6.87 (6.22–7.54)	4.63 (4.18–5.13)	5.77 (5.23–6.36)	7139.4 (6472.6–7810.8)	5048.7 (4565.3–5561.9)	6130.8 (5571.7–6733.0)	12.4 (9.8–15.3)
Australasia	2.4 (2.7–2.2)	1.4 (1.3–1.5)	3.8 (3.4–4.2)	17.26 (15.45–19.28)	10.51 (9.53–11.58)	13.99 (12.67–15.52)	10148.5 (9090.2–11415.8)	6369.5 (5779.7–7041.4)	8335.2 (7537.9–9261.7)	10.2 (7.6–12.7)
Caribbean	1.8 (2.0–1.6)	1.3 (1.1–1.4)	3.0 (2.7–3.4)	7.57 (6.82–8.43)	5.66 (5.08–6.32)	6.64 (5.99–7.37)	6421.6 (5784.5–7143.6)	5131.9 (4601.8–5708.3)	5808.8 (5244.4–6439.2)	7.9 (6.2–9.6)
Central Asia	2.7 (3.0–2.4)	1.6 (1.5–1.8)	4.3 (3.8–4.9)	5.87 (5.2–6.66)	3.82 (3.41–4.29)	4.87 (4.35–5.51)	6129.4 (5462.4–6943.4)	4722.3 (4262.2–5263.1)	5518.5 (4957.3–6181.7)	4.9 (3.7–6.1)
Central Europe	6.1 (6.7–5.5)	4.4 (4.0–4.8)	10.4 (9.4–11.5)	10.67 (9.61–11.79)	8.36 (7.56–9.25)	9.56 (8.64–10.55)	5374.2 (4854.1–5977.7)	4910.2 (4436.2–5416.3)	5165.5 (4661.3–5715.9)	7.7 (6.6–8.7)
Central Latin America	9.1 (10.1–8.2)	6.7 (6.0–7.4)	15.8 (14.2–17.5)	7.37 (6.66–8.2)	5.87 (5.28–6.52)	6.65 (5.99–7.39)	7037.6 (6346.2–7809.2)	5958.1 (5380.1–6605.2)	6536.8 (5908.8–7244.4)	11.1 (10.0–12.5)
Central Sub-Saharan Africa	1.8 (2.1–1.6)	1.4 (1.2–1.5)	3.2 (2.9–3.6)	2.82 (2.51–3.16)	2.13 (1.91–2.41)	2.48 (2.21–2.8)	5998.9 (5355.4–6713.3)	5071.1 (4576.9–5660.5)	5580.1 (5021.9–6250.8)	3.7 (1.8–5.5)
East Asia	81.7 (91.8–72.8)	55.5 (49.3–62.5)	137.3 (122.0–154.1)	11.65 (10.36–13.07)	7.84 (6.96–8.82)	9.73 (8.65–10.92)	7357.8 (6566.5–8236.3)	5239.7 (4678.5–5891.6)	6324.6 (5651.0–7078.9)	10.6 (9.3–11.8)
Eastern Europe	17.0 (19.4–15.0)	9.7 (8.7–10.9)	26.7 (23.7–30.3)	15.65 (13.84–17.85)	10.61 (9.49–11.97)	13.35 (11.86–15.15)	8397.1 (7409.8–9606.2)	7283.7 (6516.6–8207.7)	7951.4 (7061.0–9031.1)	2.4 (0.7–4.2)
Eastern Sub-Saharan Africa	5.4 (6.1–4.8)	4.3 (3.9–4.9)	9.7 (8.7–10.9)	2.66 (2.37–2.99)	2.21 (1.99–2.48)	2.44 (2.19–2.73)	5994.9 (5390.6–6709.0)	5184.0 (4684.9–5771.8)	5608.4 (5056.0–6260.2)	5.8 (5.0–6.7)
High-income Asia Pacific	22.1 (24.4–20.1)	10.1 (9.1–11.1)	32.2 (29.4–35.4)	23.97 (21.75–26.39)	11.61 (10.47–12.82)	17.97 (16.37–19.71)	10912.3 (9818.5–12134.5)	5639.9 (5084.2–6229.0)	8369.7 (7626.9–9263.7)	8.0 (6.6–9.5)
High-income North America	34.9 (38.9–31.4)	21.8 (19.6–24.4)	56.7 (51.0–63.4)	19.56 (17.6–21.82)	13.04 (11.7–14.62)	16.41 (14.74–18.34)	11264.2 (10111.1–12613.5)	7983.3 (7219.5–8914.5)	9704.7 (8749.7–10856.4)	4.1 (0.7–7.6)
North Africa and Middle East	13.3 (14.8–11.9)	11.3 (10.2–12.7)	24.6 (22.1–27.3)	4.68 (4.2–5.23)	3.81 (3.41–4.26)	4.24 (3.81–4.71)	5925.2 (5326.4–6569.0)	4786.2 (4300.3–5304.4)	5342.8 (4815.9–5907.8)	10.4 (9.2–11.6)
Oceania	0.2 (0.2–0.2)	0.2 (0.1–0.2)	0.4 (0.3–0.4)	3.4 (3.01–3.82)	2.53 (2.24–2.86)	2.96 (2.61–3.32)	5697.4 (5083.0–6347.3)	4159.9 (3701.9–4635.1)	4907.2 (4386.3–5466.5)	7.0 (4.4–9.7)
South Asia	43.9 (49.0–39.6)	31.7 (28.4–35.4)	75.6 (68.1–84.5)	5.06 (4.56–5.66)	3.57 (3.2–3.98)	4.31 (3.88–4.81)	5988.7 (5408.6–6664.5)	4435.0 (3990.0–4914.0)	5219.0 (4711.2–5790.9)	10.4 (9.4–11.2)
Southeast Asia	15.9 (17.8–14.1)	10.8 (9.5–12.1)	26.7 (23.6–29.9)	4.84 (4.29–5.42)	3.36 (2.96–3.77)	4.11 (3.63–4.6)	4633.5 (4117.8–5161.8)	3548.0 (3157.6–3959.6)	4128.9 (3676.7–4603.3)	13.0 (11.6–14.4)
Southern Latin America	4.3 (4.8–3.8)	2.3 (2.0–2.5)	6.5 (5.9–7.3)	12.98 (11.67–14.61)	7.55 (6.83–8.36)	10.38 (9.39–11.57)	9592.2 (8604.2–10784.8)	6156.8 (5563.7–6806.5)	8013.5 (7246.6–8953.5)	9.3 (7.5–11.5)
Southern Sub-Saharan Africa	2.1 (2.4–1.9)	1.7 (1.5–1.9)	3.8 (3.5–4.3)	5.48 (4.9–6.15)	4.68 (4.24–5.21)	5.1 (4.59–5.69)	6437.4 (5777.2–7194.6)	6676.9 (6058.9–7380.3)	6542.3 (5904.3–7279.5)	6.7 (5.6–7.7)
Tropical Latin America	8.6 (9.5–7.7)	5.9 (5.3–6.5)	14.5 (13.0–16.1)	7.71 (6.92–8.56)	5.71 (5.12–6.32)	6.75 (6.07–7.49)	6403.5 (5754.3–7090.1)	5237.9 (4702.7–5776.5)	5869.8 (5280.9–6498.3)	9.7 (8.5–11.1)
Western Europe	35.3 (38.9–32.2)	21.8 (19.7–23.9)	57.0 (51.9–62.9)	16.46 (14.98–18.15)	10.92 (9.9–12.03)	13.79 (12.58–15.21)	8166.6 (7396.8–9070.3)	5828.6 (5278.1–6422.3)	7056.9 (6394.9–7801.7)	6.3 (4.8–9.3)
Western Sub-Saharan Africa	6.8 (7.6–6.1)	5.2 (4.7–5.8)	12.0 (10.7–13.4)	2.97 (2.65–3.34)	2.4 (2.15–2.68)	2.69 (2.41–3.02)	6422.2 (5788.3–7164.8)	5374.8 (4850.5–5984.6)	5922.7 (5346.0–6596.3)	5.7 (5.0–6.4)

All data in this table were extracted from the following website http://ghdx.healthdata.org/gbd-results-tool

## Incidence

According to the 2017 GBD study, about 14.9 million incident cases of OA were reported globally with an age-standardized incidence rate (ASIR) of 181.2 per 100,000 person-years, an increase of 8.2% from 1990 to 2017.^13^ The ASIR of OA was highest in high-income regions, such as North America (306.6), Australasia (236), and Asia Pacific (233.4), while it was lowest in Eastern Sub-Saharan Africa (133), Central Sub-Saharan Africa (135.9) and East Asia (136.2).^13^ At the regional levels, the ASIR of OA in 2017 was highest in the U.S. (316.9), Kuwait (260.7), and Qatar (258), whereas it was lowest in Taiwan (99.5), North Korea (105.7), and Madagascar (122.3).^13^ Moreover, the U.S., Oman, and Equatorial Guinea were the top three countries with the greatest growth in ASIR of OA from 1990 to 2017.^13^ The Central African Republic and DR Congo were the only two of the 195 countries that exhibited a trend toward decline in the OA incidence.^13^ In a Chinese longitudinal cohort study, the 10-year average ASIR of knee OA was 25.2 per 1000 person-years.^27^ Data from a population-based healthcare database in England reported that the annual consultation incidence of OA was 8.6/1000 persons ≥15 years of age, 6.3 in males, and 10.8 in females.^28^ Another study estimated that the ASIR (per 100,000 person-years) was 240 for knee OA, 88 for hip OA, and 100 for hand OA.^29^ The incidence of hand, hip, and knee OA increased with age, reached the peak level at 55–59 years old, and gradually declined in the older groups.^13,29^ Women have a higher incidence of hand, foot, and knee OA, while men have a higher incidence of shoulder and cervical OA.^29,30^

## Burden

OA has been a leading cause of disability globally, which leads to enormous healthcare and socioeconomic burdens.^31^ In 2010, hip and knee OAs were among the top 20 causes of disability globally and the top 40 highest in the disability-adjusted life-years (DALYs).^32^ In 2017, OA caused nearly 9.6 million years lived with disability (YLDs), with an age-standardized rate (ASR) of 118.8 YLD per 100,000.^13^ Moreover, the age-standardized YLD was elevated by 9.6% from 1990 to 2017.^13^ Data from the 2019 GBD study showed that OA caused 18.9 million YLDs with an ASR of 244.9 (95% UI 123.7–486.7) YLDs per 100,000. The percentage changes in age-standardized YLD rates between 1990 and 2019 were 6.45% (95% UI 4.84% to 7.96%). OA is estimated to contribute $460 billion in all-cause medical costs in 2019 in the United States.^33^ It was also reported that OA patients had four times excess total medical costs than people without OA ($14,521 vs. $3629 per year per person).^34^ The 2015 GBD study analyzed 315 causes of diseases and their burdens. OA ranked as the 15th leading cause of YLDs, contributing a total of 52,661 (UI 34,056–77,499) YLDs in the Nordic region in 2015.^35^ In China, the YLDs caused by knee OA were 4,149,628, and the YLDs per 100,000 was 968. Moreover, the total YLDs caused by OA was 1.97 million in 2017, accounting for 1.08% of all YLDs.^36^ Currently, the burden of OA is still continuously increasing in most countries.^16,37^ Risk factors in OA etiology, such as female gender and advanced age, are correlated with high YLD rates.^38^

## Risk factors

Risk factors of OA include obesity, female gender, aging, knee injuries, and high-impact sports, such as marathons, speed skating, and weightlifting. Although aging and OA could be totally independent processes, they are closely associated from a statistical point of view.^39–41^ Aging has been accepted as the most prominent risk factor for OA. Except for increasing exposure to other risk factors with age, aging-related biological and molecular signaling alterations significantly contribute to the disorganization of the joint structure. Several potential pathological mechanisms of how aging contributes to OA have been proposed recently.^41,42^ The cellular senescence-associated secretory phenotype (SASP) has been detected in the degradative articular cartilage and synovial joint tissue of developing OA.^43–45^ In addition, aging-related mitochondrial dysfunction that induces oxidative stress, characterized by excessive accumulation of the reactive oxygen species (ROS) with the imbalance of energy metabolism of articular chondrocytes, is believed to promote articular chondrocyte apoptosis and articular cartilage destruction.^46–48^ Furthermore, age-related inflammation in the synovial joint, which is also associated with SASP, leads to destructive changes in the extracellular matrix (ECM) of the articular cartilage and promotes OA.^49^

Obesity is another major risk factor that leads to a higher incidence of hip and knee OA. Obesity is one of the most significant risk factors for knee OA partly because the excessive weight of obese patients leads to an abnormal increase in mechanical loading on knee joints, which results in the wearing and tearing of articular cartilage accompanied by ligament destruction and eventually leads to the occurrence of OA. Surprisingly, obese patients also have a higher incidence of OA in the hands that do not usually bear the weight.^50,51^ This leads to the general belief that it is the systemic factor(s) released by other tissues that induce OA in obese patients. In obese patients, cytokines released by adipocytes, also known as “adipokines”, such as resistin, visfatin, leptin, omentin, adiponectin, retinol-binding protein 4 (RBP4), and other factors, were reported to be associated with promoting the initiation and progression of OA.^52–56^ Furthermore, cytokines, such as TNF-α, IL-1, IL-6, and IL-8, were shown to trigger joint inflammation, which leads to ECM breakdown and cartilage degeneration.

OA affects more than 500 million populations worldwide, with a higher prevalence in the female gender than in males. Women are known to be more susceptible to OA onset and development than men are.^6,57,58^ Several studies showed that OA development could be triggered by the plunge in sex hormone levels in menopausal women.^59,60^ Besides, compared to male OA patients, female patients were reported to have higher levels of joint inflammation and clinical pain, thinner articular cartilage, and severe physical joint mobilities.^57,60,61^ The potential contributing factors for this gender difference in OA are not fully understood and need further attention in the OA research community.

Knee injury is another major risk factor for knee OA. Post-traumatic OA is one of the OA subtypes that occurs in those joints that have been injured. Current studies have shown that joints that have been traumatized are five times more likely to develop OA than joints that have never been damaged.^62^ U.S. clinical statistics predict that post-traumatic OA accounts for 12–42% of OA (the proportion varies by age), and the actual proportion could be higher.^3^ Trauma in the joints has been demonstrated to induce massive gene expression alterations in different compartments of knee joints. Besides injury, sports-related excessive joint loading also increases the chance of OA development. Professional athletes of high-impact sports present a higher prevalence of early knee OA than non-professional athletes and the universal population.^63^ The new technology of instrument innovations has been developed and utilized for investigating the role of joint mechanical stress in OA pathogenesis, which could study the pattern, force, and duration of mechanical stress on joint loading.^64,65^ Our advanced knowledge of how mechanical loading contributes to OA onset and progression is just beginning to be used in the applications of biomechanics assessment for guiding the clinical physical therapy of OA patients.^66^ However, the molecular mechanisms of how mechanical stress contributes to OA onset and development need to be investigated in great detail.

In comparison, few genetic mutations have been confirmed to be linked to human OA before. It is until recently that genetics has been discovered as a risk factor in 11 types of OA, including OAs in the hand, hip, and spine.^67^ A recently reported genome-wide association study (GWAS) meta-analysis of more than 820,000 East Asian and European individuals from 13 international cohorts of 9 populations, including over 170,000 OA patients, identified around 10,000 significantly associated single-nucleotide variants (SNVs), in which 100 were unique and showed independent genetic correlation with OA phenotypes and symptoms.^67^ Among these identified 100 SNVs, 60 were genome-wide significantly associated with more than one type of OA, and 77 potential effector genes were identified. Though genetic studies identified risk variants associated with new molecular signals and already reported effector genes contributing to the OA development,^68–71^ these genetic risk data need to be further verified and investigated to reveal options for the translational intervention of OA.

## Clinical symptoms

Clinical symptoms of OA include joint functional limitations, stiffness, pain, disability of walking or running, and probably other symptoms.^1,72,73^ Bony enlargement and swollen and inflamed joints could be found in OA patients in physical examination. Clinical radiographic examination, such as MRI (magnetic resonance imaging), is able to visualize marginal osteophytes, joint space narrowing, structural changes of osteochondral tissue, and other OA lesions. Pain is one of the most distinctive symptoms and the main reason OA patients seek medical help,^72^ but the underlining mechanisms of OA pain are still poorly understood. Pain is a clinical indicator of tissue damage, inflammations, or disorders of the nervous system.^74,75^ Articular cartilage is an avascular tissue without any nerve invasion, and OA pain can happen both before and after an articular cartilage lesion detected by the imaging system. Therefore, it is unlikely that the destruction of articular cartilage directly causes OA pain. OA pain has been reported to be associated with synovitis and bone marrow lesion,^76,77^ as well as alterations in subchondral bone, osteophyte formation, abnormalities of infrapatellar fat pads, and lesion of ligaments, in which tissues have highly distributed sensory nerves.^78^ Molecular mechanisms underlying OA pain have been comprehensively updated and discussed in two recent review articles.^79,80^

## Pathogenesis

During the past decades, the pathogenesis of OA has been extensively studied.^81^ Although its risk factors were characterized, and the structural changes of the synovial joint in OA are well understood, the complex pathological mechanisms of the onset and development of OA remain elusive. We summarize our current understanding of OA pathogenesis from the perspective of tissue interactions, changes in cellular biology, and pathogenic signaling pathways and molecules.

### Tissue interactions in OA

Numerous studies reported that subchondral bone sclerosis could be one of the major reasons that cause aging-related OA and that the abnormal bone remodeling related to dysregulation of osteoblasts and osteoclasts plays key roles in the OA initiation and development.^82–87^ Increased subchondral bone porosity and remodeling, reduced bone density, and bone mineralization with irregular matrix organization, which were believed to be stimulated by bone-cartilage crosstalk through subchondral pores and vascular invasion, were observed in the early stage of OA.^88–91^ These changes in subchondral bone were found to be happening at the same time with or earlier than the early destruction of articular cartilage.^92–95^ On the other hand, the late stage of OA showed architectural alterations of the subchondral bone characterized by a reduction of bone remodeling and enhanced subchondral bone densification leading to sclerosis.^96–98^ However, the mechanism of how articular cartilage and joint issues crosstalk with subchondral bone leading to the initiation and development of OA is incompletely understood and needs further investigation.

Besides the subchondral bone dysregulation, the synovium is another most-related tissue that showed significant changes at the early stage of OA, even before cartilage degradation occurs.^91,99^ The contribution of synovium to the initiation and development of OA has been investigated in the past 20 years. At the early stage of OA, histological changes of the synovium include synovial lining hypertrophy and hyperplasia, increased angiogenesis, a low level of synovial inflammation, and synovial fibrosis observed.^100–103^ Synovitis with a high level of macrophages could be found at the end stage of the OA.^104^ Synovitis scores used as one of the OA assessments are based on these histological features.^99,105,106^ Low-grade synovial inflammation can be detected in >50% of OA patients at the early and late stages of the disease.^107,108^ Therefore, among the synovial features, synovial inflammation has received the most attention from the OA research community. It is widely believed that pro-inflammatory factors that are released by synovial tissue induce the ECM destruction of the articular cartilage. However, the interactions of different cell types in the synovial joint and features of the synovium at different stages of OA need to be extensively investigated.

Obesity acts as an OA risk factor not solely through loading excessive body weight onto knee joints,^109^ the pathogenesis involves a complex network of tissue and cellular interactions. As mentioned above, adipokines released by adipose tissue that interact with different tissues are believed to be critically involved in OA pathogenesis.^110–112^ In addition, the local adipose tissue, such as the infrapatellar fat pad (IPFP), also cross-talks with other synovial tissues to affect OA development.^111,113^ Recently, a completely fat-free transgenic lipodystrophy (LD) mouse model, which has a comparable body weight to wild-type controls, was used in an OA study to demonstrate the contribution of adipose tissues to OA onset and development. LD mice with a total lack of adipose tissue are resistant to DMM-induced or spontaneous OA. The susceptibility to posttraumatic OA was restored after mature fat depots were implanted in the LD mice, suggesting the notion that the adipose tissue and factors released by it can promote OA lesions.^114^ On the other hand, adipose tissue could be used as a good source of heterogeneous cell populations, including lymphoid, adipose-derived stromal/stem cells, and myeloid cells and scaffolds, for OA treatment.^115^

### Cell biology of the articular cartilage

Cellular changes of different cell types of synovial joint tissue in OA have been discussed in several excellent review articles.^1,116^ Although tissue interactions are important in the onset and progression of OA, the destruction of articular cartilage is a hallmark of the majority of OA cases, especially knee OA. Chondrocytes are the only cell type in articular cartilage. Chondrocytes in the degradative articular cartilage undergo more active cell death than those in normal articular cartilage. Apoptosis, a highly regulated programmed cell death, is the earliest reported cell death form.^117^ While it is known that a low level of apoptosis detected by TUNEL assay exists in normal articular cartilage, the number of apoptotic cells is dramatically increased in the early and late stages of OA articular chondrocytes. Several studies have shown that death receptors (e.g., FAS, TNFR),^118,119^ cytokines (e.g., TNF-α, IL-1β),^120–122^ and abnormal mechanical stress and physicochemical factors, including ultraviolet radiation and ionizing radiation, can cause chondrocyte apoptosis.^123^ In addition, three signaling pathways have been shown to induce apoptosis of articular chondrocytes in OA: the mitochondrial-mediated caspase-dependent pathways, the death receptor pathway, and the endoplasmic reticulum stress (ER stress)-induced unfolded protein response (UPR) pathway. However, whether apoptosis is the cause or consequence of OA remains to be determined. Besides apoptosis, autophagy has been considered to be involved in combination with apoptosis in contributing to OA development.^124^ While autophagy is activated at the early stage of OA to promote cell survival, it facilitates cell death at the end stage of OA. Ferroptosis is a newly discovered type of cell death driven by iron-dependent lipid peroxidation and characterized by excessive accumulation of lipid ROS.^81,125^ Pyroptosis, which can be triggered by proinflammatory factors, was recently shown to promote chondrocyte death and OA.^126^

The superficial layer accounts for 10–20% of the total thickness of the articular cartilage, and chondrocytes in this layer are flat and have a relatively high-density,^127^ while the chondrocytes in the middle and deep layers are relatively sparse. Chondrocytes, which are large and round in shape in the deeper layer, are divided and differentiated by chondrocytes from the superficial layer.^128^ OA is characterized by the increasing production of ECM-degrading enzymes and the removal of articular cartilage, which is very similar to the process of endochondral ossification, and abnormally induced articular chondrocyte hypertrophic differentiation is one of the key factors that promote the onset and progression of OA. Thus, factors that accelerate this process are probably important risk factors for inducing OA. However, the mechanisms that regulate articular chondrocyte differentiation are poorly defined. It is known that the ultimate differentiation process and cell function of chondrocyte hypertrophy is regulated by a variety of in vivo systemic factors and local signaling factors, including growth hormone, insulin-like growth factor, parathyroid hormone, parathyroid hormone-associated protein, Indian hedgehog, fibroblast growth factor, classical and non-classical Wnt signaling pathways, transforming growth factor families, RUNX2 (Runt-related transcription factor 2) and MEF2 (myocyte enhancer factor 2) transcription factors, etc.^129–131^

**Fig. 2:**
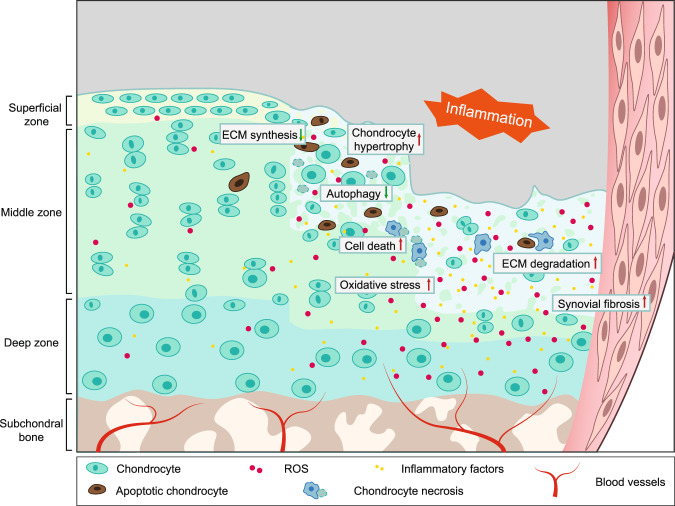
Histological characteristics of OA articular cartilage. Articular cartilage is composed of a dense extracellular matrix (ECM) and sparse distribution of chondrocytes. A cross-sectional diagram of articular cartilage can be divided into the superficial zone, middle zone, and deep zone (the lower part is calcified). Chondrocytes and ECM in the OA cartilage are extensively changed. Cell apoptosis and necrosis are increased in OA chondrocytes with inhibited anabolism and boosted catabolism leading to suppressed ECM synthesis and enhanced ECM degradation. Inflammatory factors, such as TNFα secreted by chondrocytes and synovial membrane fibroblasts, stimulate inflammation in the whole joint. Synovial hyperplasia and fibrosis, and abnormal blood vessel invasion from subchondral bone is observed in OA synovial joint. Increased chondrocyte hypertrophy and oxidative stress with excessive ROS production are reported

In the past decades, more and more evidence suggests that metabolism plays a crucial role in maintaining the energy homeostasis of articular chondrocytes and that abnormalities in chondrocyte metabolism can lead to the onset and progression of OA.^132,133^ Abnormally accelerated catabolism of articular chondrocytes, which promotes ECM degradation and overtakes ECM synthesis, is a major feature of the OA cartilage.^132^ With the low-grade inflammation in the synovial membrane and abnormal blood vessel invasion through the subchondral bone, the cellular hypoxic stress, oxidative stress, and autophagy level of synovial membrane cells and articular chondrocytes are constantly changing and interplaying with each other, thus contributing to the pathogenesis of OA (Fig. 2).

## Pathogenic pathways and molecules

### Wnt signaling

Wingless-like (Wnt) glycoproteins are a family of more than 19 secreted proteins, which are poorly soluble molecules due to their lipid sidechains. The reduction of solubility of Wnt ligands strongly limits their physical signaling transduction capacity in different tissues and typically generates concentration gradients impacting adjacent cell behavior.^134,135^ With both paracrine and autocrine effects, Wnt signaling was demonstrated to play an essential role in skeletal development in terms of mesenchymal stem cell condensation, chondrogenic differentiation, chondrocyte hypertrophy, growth plate chondrocyte organization, and osteoblast differentiation and maturation.

**Fig. 3:**
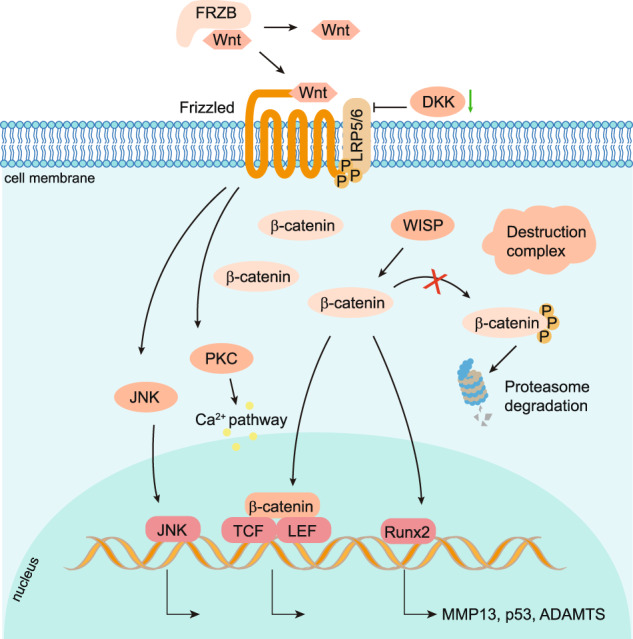
Activated Wnt canonical and non-canonical signaling pathways in OA. In healthy articular cartilage, the Wnt signaling pathway in articular cartilage and synovium is profoundly inhibited by the absence of Wnt-Wnt receptor interactions and β-catenin degradation through the proteasome pathway. In OA chondrocytes and synovial membrane cells, Wnt ligands bind to Frizzled/LRP5/6 receptors to activate the canonical pathway, in which the destruction complex moves to the cell membrane and releases β-catenin to translocate to the nucleus and binding with TCF/LEF family to control target gene transcription. For the non-canonical Wnt pathway, the protein kinase C (PKC) and c-Jun N-terminal Kinase (JNK) are activated to upregulate downstream target genes and pathways, including the calcium pathway

Wnt pathways are classified into two types by their dependency on β-catenin (Fig. 3): the canonical pathway that is β-catenin-dependent and the non-canonical pathway that is independent of the β-catenin signaling.^136^ β-catenin is a subunit of the cadherin protein complex and the co-activator to promote its downstream target gene transcription.^137^ In the cytoplasm, β-catenin binds to cadherin protein on the cell membrane attaching to cytoskeleton protein actin and participating in cell adhesion.^138^ After being phosphorylated by GSK3β, β-catenin forms a complex with GSK3β and is degraded through ubiquitination.^138^ In the canonical pathway, Wnt ligands bind to Frizzled and co-receptor LRP5/6 (low-density receptor-related protein 5 or 6), which then recruits protein complex to inhibit the proteasomal degradation and promote nucleus translocation of β-catenin to transcribe Wnt target genes.^139–141^

Modest Wnt canonical signaling activation is necessary for maintaining healthy articular cartilage. Inhibiting the activity of β-catenin in articular chondrocytes to inactivate the canonical Wnt pathway by overexpressing a small molecule ICAT (Inhibitor of β-catenin and T cell factor) in Col2a1-ICAT transgenic mice, caused articular cartilage degradation with enhanced chondrocyte apoptosis.^142^ In addition, mice with global knockout of Wnt16, which is dramatically upregulated in articular cartilage of OA patients and mouse models, developed a more severe articular cartilage degradation than wild-type mice with increased chondrocyte apoptosis and reduction of lubricin expression after induced destabilization of the medial meniscus (DMM).^143^ Wnt16 alleviates the progression of OA by reducing cartilage catabolism through the planar cell polarity (PCP)/JNK-mTORC1-PTHRP cascade and inhibiting the chondrocyte hypertrophy.^144^ Most Wnt ligands play non-redundant crucial roles during mouse embryogenesis.^145^ Currently, there are few genetic mouse models with Wnt genes knocked out in articular chondrocytes that develop spontaneous OA. Of note, the excessive Wnt signaling might promote the development of OA. β-catenin is significantly upregulated in the OA mouse superficial articular chondrocytes and increased in the cartilage of OA patients. The polymorphisms in the frizzled-related protein (FRZB) gene, which is a secreted Wnt antagonist, showed an association with hip OA in females.^146^ Though they do not develop spontaneous OA nor stabilize more β-catenin in the articular chondrocyte, *Frzb* global knock-out mice exhibited increased cartilage loss with upregulation of MMP3 in the mouse arthritis models induced by instability and inflammation.^147^ Activating the Wnt signaling by mutating the three GSK3β phosphorylation sites within the β-catenin molecule in chondrocytes using the *Col2a1-Cre* transgenic mice led to the embryonic lethetic phenotype.^148–150^ However, inducible activation of the β-catenin in chondrocytes of adult mice using the *Col2a1-Cre*^*ERT2*^ transgenic mice caused dramatic destruction of the articular cartilage and other OA-like phenotypes in the knee, hip, and TMJ joints.^5,151–154^ Moreover, a recent study showed that activating β-catenin by injection of LiCl in the synovial joint of rabbits promoted cartilage degeneration, and the increase of MMP-13 and P53, leading to cartilage degradation and OA development.^155^ All these lines of evidence indicate that the overactivation of β-catenin in articular chondrocytes promotes the initiation and progress of OA. Interestingly, activating β-catenin signaling by expressing a constitutively active form of β-catenin under the control of the cartilage-specific *Col11a1* promoter in chondrocytes in adult mice induces early closure of the growth plate and leads to significant increases in thickness, cell proliferation rate, and cell density, especially in the superficial layer of the articular cartilage.^156,157^

To extensively understand the fundamental role of Wnt signaling in the articular cartilage and the development of OA, more animal models with genetic manipulation of Frizzled (Fz) receptor proteins, single Wnt ligands, and β-catenin in the articular cartilage should be studied. Wnt signaling pathway-related genes are more extensively dysregulated in the synovium than in the articular cartilage in the STR/Ort mice and collagenase-induced OA mouse models, especially at the early stage of OA.^158^ In the articular cartilage, the expression of Frizzled receptors generally, especially Fz6, responded to OA initiation much earlier than detectable changes of Wnt ligands in mouse OA models,^158^ suggesting that Wnt signaling might initially have the paracrine effects in the early stage of mouse OA. Dickkopf-1 (DKK1, Wnt antagonist) levels in plasma and synovial fluid of OA patients were significantly reduced,^159^ and the level of DKK1 were negatively associated with the severity of OA.^160^ Overexpression of Dkk1 but not Dkk2 using *Col2a1-Cre* in chondrocytes inhibited the articular cartilage damage induced by DMM surgery in mice.^161^ Overexpression of Dkk1 in bone cells using *Col1a1-Cre* induced fewer cartilage lesions in OA mouse models.^161,162^ The expression of Dkk3 was significantly increased in the human OA cartilage, synovial tissue, and synovial fluid.^163,164^ However, the role of Dkk3 and Dkk4 in the onset and progression of OA needs to be determined. In the synovium of OA patients, multiple Wnt family proteins are overexpressed^165^; however, their potential mechanisms in synovial inflammation are still unclear. WISP-1 (WNT1-inducible-signaling pathway protein 1) significantly increased in the synovium and articular cartilage in human OA and experimental mice OA. Overexpressing recombinant WISP-1 in synovium and articular chondrocyte stimulated macrophages and chondrocytes, causing interleukin-1 to independently induce several aggrecanase and matrix metalloproteinases (MMPs), and the overexpression of MMPs and aggrecanase in joints caused articular cartilage damage.^158,160^ In the non-canonical pathway, Wnt ligands bind to frizzled, and the co-receptors, PKC (the protein kinase C) and JNK (c-Jun N-terminal Kinase) are then activated to upregulate downstream target genes and pathways, including calcium pathway. These two pathways are both involved in the onset and development of the OA.^141,166^ Wnt 5a and Wnt11 are the two ligands that are associated with the non-canonical pathway, and they are both detected in the human OA cartilage.^167^ The role of the Wnt non-canonical pathway in OA development remains to be identified.

Since overactivation of Wnt signaling is associated with OA development, the Lorecivivint (SM04690),^168^ the first inhibitor of the β-catenin-related pathway, was subjected to the phase I clinical trial as a disease-modifying osteoarthritis drug (DMOAD) in 2015,^169,170^ and has recently entered phase III clinical trial (NCT05603754) in Nov. 2022 as an OA-modifying agent. SM04690 was shown to induce the bone-marrow-derived human mesenchymal stem cells to chondrocyte differentiation and promote regeneration and protection of articular cartilage in vivo and in vitro through inhibiting CLK2 and DYRK1A, thus leading to the inactivation of β-catenin associated pathway.^168,169,171^ Intra-articular injection of other small-molecule inhibitors of Wnt/β-catenin, such as XAV-939 and C113, alleviated OA phenotypes in terms of decreased articular cartilage degradation and synovitis in vivo.^172^ Several other Wnt/β-catenin inhibitors have been investigated in cultured cells and animal models.^173–176^ However, when the Wnt/β-catenin pathway was excessively inhibited, proliferation and hypertrophy of growth plate chondrocyte were disrupted, the formation of secondary ossification center was delayed in long bone, and articular cartilage destruction occurred.^177^ Therefore, other β-catenin-independent Wnt pathway-related strategies need to be investigated and developed. Injecting Ad-Wnt16 into the joint to activate the Wnt16 pathway significantly reduced the symptoms of OA.^144^ This may provide new ideas for the design of DMOAD.

### NF-κB pathway

Nuclear factor-κB (NF-κB) constitutes a group of transcription factors in which the pathways can be activated by different kinds of pro-inflammatory cytokines.^178^ NF-κB plays a critical role in the inflammation, differentiation, proliferation, and survival of mammalian cells.^178,179^ The NF-κB family contains five members: RelA/p65, RelB, c-Rel, NF-κB1/p50(p105), and NF-κB2/p52(p100). These five proteins all contain an N-terminal Ref-1-homology domain, which is evolutionally conserved and contributes to the dimerization of protein complexes, nuclear localization, DNA banding, and interaction with NF-κB inhibitors.^180^ The NF-κB is a homo- or hetero-dimeric complex which can be divided into two categories according to its subunit combination.^181^ One category includes p50 and/or p52 which are processed from their precursor p105 (NF-κB1) and p100 (NF-κB2),^182^ respectfully. The second category includes c-Rel, p65, and Rel B, which contain a transcriptional trans-activation domain (TAD).^182^ Lacking TAD, the dimers formed only by p50 and p52, either homodimers or heterodimers, could not drive the transcription.^181^ In contrast, the heterodimers of c-Rel:p50, p65:p50, and RelB:p52 could function as transcriptional activators.^181^ In eukaryotes, two classic dimers (p65:p50 and RelB:p52) are the most common NF-κB complexes that work as transcriptional activators.^181^ Inhibitors of NF-κB (IκB) proteins, including IκBα, IκBβ, IκBγ, IκBε, IκBζ, and Bcl-3, bind to NF-κB family members in the cytoplasm to block phosphorylation and activation of NF-κB.^183,184^ A variety of inflammatory signals, such as TNFα, can activate the IκB kinases (IKKs), and the activated IKKs lead to IκB degradation by regulating its phosphorylation.^183^ Then, the NF-κB complex translocates to the nucleus, where it triggers the transcriptions of downstream target genes.^185^

The expression of NF-κB proteins varies in different stages of cartilage development.^186^ p65 is expressed in the entire growth plate, especially highly expressed in hypertrophic chondrocytes in vivo and in vitro, suggesting that the NF-κB pathway participates in the endochondral ossification.^186,187^ Over-expression of IκBα blocks NF-κB activation, thereby inhibiting the Rel/NF-κB protein expression and leading to abnormal development of limb bud in chicken embryos.^188^ In turn, activation of the NF-κB pathway promotes the proliferation and maturation of chondrocytes by treating with IGF-1 in vitro.^189^ Deleting p100 in mice resulted in dwarfism, primarily caused by impaired chondrocyte hypertrophy. However, these defects were partially rescued when the RelB gene was knocked down in *p100*^*−/−*^ mice.^190^ Using tissue-specific knockout mice, Kobayashi, H. et al. found that Rela/p65 deletion in embryonic limb cartilage promoted apoptosis of chondrocytes leading to impaired skeletal growth, while homozygous knockout of Rela in articular cartilage at seven weeks resulted in accelerated progression of arthritis, but heterozygotes knockout led to delayed OA development by inhibiting the expression of catabolic genes.^191^

**Fig. 4:**
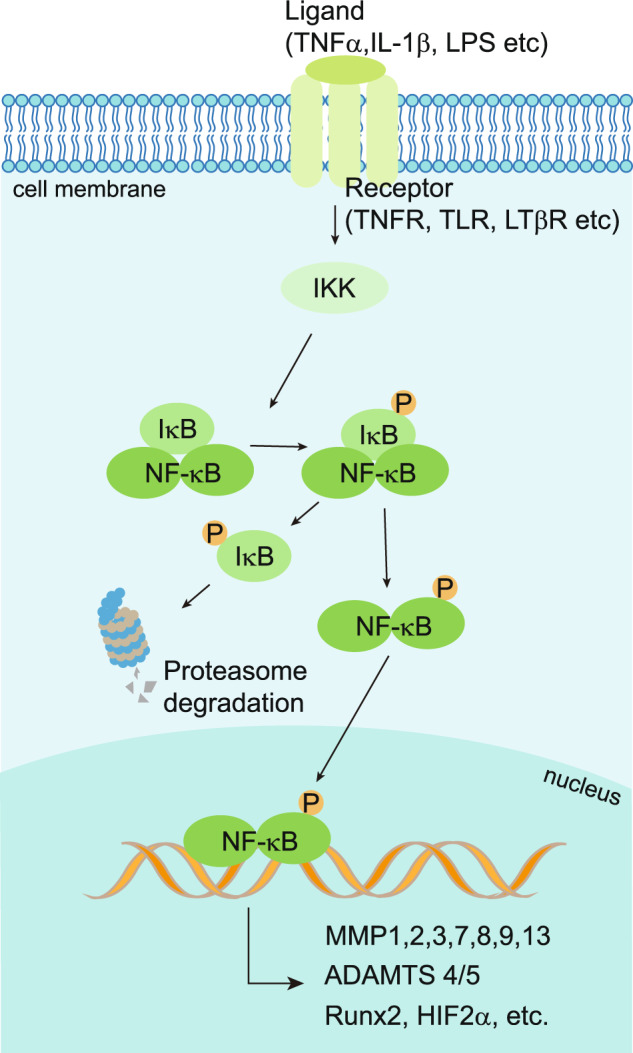
The activity of NF-κB signaling in OA chondrocytes. The NF-κB signaling is activated by a variety of ligands, including TNFα, IL-1β, and LPS, leading to phosphorylation (P) and proteasome degradation of IκB. Receptors of NF-κB signaling include TNFR, TLR, LTβR and etc. Activated IKKs lead to IκB degradation by regulating its phosphorylation. Thus, the translocation of NF-κB to the nucleus results in the transcription of the downstream target genes encoding RUNX2, MMPs, ADAMTS, and HIF2α

NF-κB signaling is extensively involved in OA pathology through a variety of patterns (Fig. 4). Mechanoreceptors, cytokine receptors, TNFR, and TLR are located on the surfaces of the articular chondrocyte cellular membrane. Activation of these receptors by pro-inflammatory mediators (such as TNFα or IL-1β), fibronectin fragments, and mechanical stress induces the NF-κB signaling^192,193^ and cross-talk with BMP (bone morphogenetic protein), Wnt, and other signaling cascades as well.^192^ NF-κB signaling induces the secretion of degrading enzymes, such as matrix metalloproteinases (MMP) (MMP1, MMP2, MMP3, MMP7, MMP8, MMP9, MMP13), ADAMTS4 and ADAMTS5, leading to degradation of articular cartilage.^192^ Additionally, numerous NF-κB-mediated cytokines and chemokines, including receptor activators (RANKL) of TNF-α, IL-1β, IL-6, and NF-κB (RANK) ligands expressed in OA articular chondrocytes could upregulate MMP production, reduce the cellular synthesis of collagen and proteoglycan, and act in a positive feedback loop to enhance NF-κB signaling.^194^ NF-κB can enhance joint injury by inducing PGE2 (prostaglandin E2), NOS (nitric oxide synthase), NO (nitric oxide), and COX2 (cyclooxygenase-2), thereby promoting tissue inflammation, synthesis of catabolic factors, and apoptosis of articular chondrocytes.^192^ NF-κB promotes the activation of other transcription factors, such as hypoxia-inducible factor 2α (HIF-2α), E74-like factor 3 (ELF3), and RUNX2.^195–198^ These transcription factors trigger the expression of ADAMTS5 and MMP13 proteases, which promotes the differentiation of pre-hypertrophic articular chondrocytes to terminal differentiated chondrocytes.^199^ In addition, Deng and coworkers found that inflammatory cytokines regulated TAK1-mediate-phosphorylation to promote proteasomal degradation of YAP (Yes-associated protein), which is a downstream regulating factor of the Hippo pathway.^200^ Activation of YAP in the articular chondrocytes inhibited substrate accessibility of TAK1, thus attenuating the NF-κB signaling.^200^ Therefore, the interactions of the Hippo-YAP pathway and NF-κB signaling regulate ECM degradative enzymes to control articular cartilage homeostasis.^200^

NF-κB signaling has been well accepted as a therapeutic target of OA.^201,202^ Thus, a series of drugs aiming at inhibiting the NF-κB signaling pathway have been developed. For example, the COX-2 isomer is regarded as a proinflammatory enzyme induced by inflammatory stimulation, and COX-2 is responsible for the production of pro-inflammatory PGE2.^201^ Glucocorticoids are also used to treat inflammatory diseases. It has been suggested that glucocorticoid treatment can not only up‐regulate the NF‐κB inhibiting protein like I‐κB but also prevent DNA binding and transcriptional activation of NF‐κB.^203^ Furthermore, receptor antagonists, such as TNF receptor inhibitors, IL‐1β receptor antagonists, IKK inhibitors, and inhibitors of NF‐κB nuclear translocation also considered novel therapeutics for the treatment of OA. The TNF inhibition strategy has recently been tested in two clinical trials. The results showed that anti-TNF antibody adalimumab and etanercept (TNF inhibitor) treatment for 12 weeks and 24 weeks, respectively, showed no differences in pain control in hand OA.^204,205^ The results of clinical trials for IL-1 inhibitors seem to be controversial. Both AMG108, a monoclonal antibody that blocks IL-1α and IL-1β, and ABT-981, an immunoglobulin targeting both IL-1α and IL-1β, displayed negative results in amelioration of the OA symptoms in patients,^204,206^ but IL-1 inhibitor canakinumab, which is used to treat atherosclerotic disease to reduce cardiovascular risk, showed a reduced rate of joint replacement cases in the treatment group.^77^ Biological treatment of OA by knee joint intra-articular injection of the anti-inflammatory drug canakinumab has been under the clinical trial phase II since Nov 2022 (NCT04814368).

### AMPK pathway

**Fig. 5:**
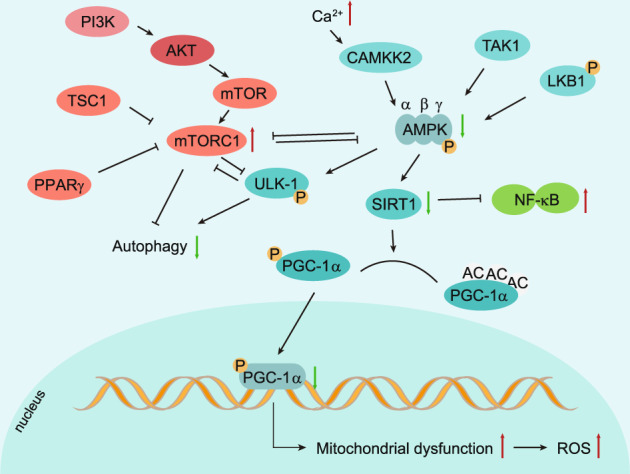
Regulation of chondrocyte autophagy and metabolism by AMPK and mTOR signaling pathways. Adenosine monophosphate (AMP)-activated protein kinase (AMPK) is a heterotrimeric complex that contains subunits α, β, and γ. LKB1 (liver kinase B1), CAMKK2 (calcium-sensitive kinase) and TAK1 (transforming growth factor-β-activated protein kinase-1) are three major enzymes that can introduce AMPK phosphorylation. In OA chondrocytes, inhibited AMPK phosphorylation decreases ULK1 expression, which is an essential initiator of autophagy. Decreased phosphorylation of AMPK also downregulates the expression of PGC-1α through SIRT-1 in articular chondrocytes, leading to mitochondrial dysfunction

Adenosine monophosphate (AMP)-activated protein kinase (AMPK) is a heterotrimeric complex that contains one catalytic subunit α and two regulatory subunits β and γ,^207^ and each subunit is encoded by two or three different genes leading to 12 different α/β/γ combinations of distinct AMPK complexes (Fig. 5). It senses low cellular ATP levels and responds to the decreasing of ATP to AMP ratio by regulating metabolic enzymes to boost ATP generation and suppress ATP consumption, which is an essential regulator for energy homeostasis. When cellular energy is low, Thr-172 in the α subunit is phosphorylated, leading to the activation of the AMPK complex. The major kinases that catalyze this reaction are the LKB1 (liver kinase B1) and calcium-sensitive kinase CAMKK2 (also known as CAMKKβ) which respond to calcium flux. AMPK expression and potent phosphorylation of AMPKα are detected in normal articular chondrocytes. Compared with normal joint tissues, the articular cartilage of OA patients and joint tissues of aging-related and surgically-induced OA mice have significantly reduced AMPKα phosphorylation at T172.^208^ Several studies have demonstrated that the expression of AMPK is significantly reduced in mouse OA articular chondrocytes and human knee OA chondrocytes.^209,210^ AMPKα phosphorylation was also significantly reduced in a DMM-induced mouse model of OA and acute gouty arthritis (AGA) rats.^208,211^ In line with these findings, deletion of AMPKα1, AMPKα2, or both in mouse cartilage caused more severe knee injury 2–4 weeks after DMM surgery,^212^ suggesting AMPK activation is a protective factor for articular cartilage. However, how the expression and activation of AMPK are reduced in OA chondrocytes is still unknown. Reduced LKB1 activity could be one of the reasons why the phosphorylated AMPK is decreased in human and mouse OA chondrocytes since downregulation of LKB1 and AMPK activity has been found in biomechanically induced OA to modulate matrix catabolism,^208^ but genetic studies on the role of LKB1 in the onset and development of OA is still missing. Interestingly, CaMKK2, another kinase that induces AMPK phosphorylation, has been reported to be elevated in DMM-induced OA cartilage and IL-1β-treated articular chondrocytes. Suppression or loss of CaMKK2-protected DMM-induced cartilage destruction.^213^ This discrepancy might be due to the other potential functions of CaMKK2 that are independent of AMPK or CaMKK2 not being the major enzyme fulfilling the AMPK phosphorylation in the articular cartilage context.

TAK1 (transforming growth factor-β-activated protein kinase-1) was another enzyme that can introduce AMPK phosphorylation in certain cellular contexts in vitro.^214,215^ Over-expressing Tak1 by intra-articular injection of adenovirus expressing TAK1 induced cartilage destruction in rats, and inhibiting TAK1 by small molecule 5Z-7 protected against DMM-induced cartilage degradation.^216^ However, this study did not examine the expression level and activity of AMPK. Therefore, whether TAK1 is one of the enzymes that phosphorylate and activate the AMPK pathway and the effect of AMPK overactivation in the articular cartilage context in vivo remains to be determined.

Dysfunction of mitochondria and impaired mitochondrial biogenesis in OA chondrocytes have been determined to be related to abnormal AMPK activity during OA development.^208,217^ AMPK activator, A-769662, which induces the upregulation of peroxisome proliferator-activated receptor-gamma coactivator 1α (PGC1α) could reverse the impaired mitochondria biogenesis in OA chondrocytes through regulating SIRT1 signaling.^209,218^ Impairment of AMPK activity further induced mitochondrial dysfunction resulting in insufficient energy supply and excessive ROS production.^208^ Furthermore, it was reported that NF-κB signaling was directly or indirectly regulated by AMPK as an important inflammatory response pathway. AMPK deacetylated p65 through SIRT1, induced proteasomal degradation of p65, and ultimately inactivated the NF-κB signaling.^219^ Activation of AMPK with a glycolysis inhibitor 2-deoxyglucose (2-DG)) reduces synovial inflammation and joint damage primarily by inhibiting the NF-κB pathway in adjuvant arthritis (AA) rats.^220^ AMPK phosphorylated and activated the ULK1(Unc-51-like kinase 1), which is a key initiator of the autophagy.^221^ AMPK also plays a key role in the regulation of ER stress. CHOP (C/EBP homologous protein), one of the UPR components, inhibited AMPK activity via IL-1β in OA cartilage where UPR activation is triggered, and activation of AMPK by AICAR (5-Aminoimidazole-4-carboxamide ribonucleotide) attenuated the enhanced expression of CHOP in biomechanical injured articular cartilage.^222^ Quercetin was reported to suppress ER stress by activating the SIRT1/AMPK signaling pathway.^223^ AMPK is involved not only in the degradation of articular cartilage but also in the pathological changes of the synovium. AMPK is involved in the anti-inflammatory response of the synovium. For example, transforming growth factor-β1 (TGF-β1) activates AMPK to promote FOXO3 expression, which inhibits the synthesis of inflammatory factors in the synovial fibroblasts of human knee OA.^224^

Restoring AMPK activity may be able to reduce inflammation and prevent OA progression. For example, the catabolic response of chondrocytes to inflammatory cytokines was significantly inhibited by the selective AMPK agonist AICAR.^209^ Metformin was reported to limit OA by upregulating the expression of total and phosphorylated AMPK in articular cartilage tissue in mice and rats.^225–227^ Further experiments demonstrated that metformin could upregulate AMPKα1 expression and delay the development of OA.^225–227^ In addition, metformin could also relieve OA pain by upregulating the expression of total AMPKα1 in DRG cells and inhibiting the pain sensitivity caused by DMM surgery.^225,228,229^ In addition, active vitamin D reduced inflammation by triggering the AMPK-mTOR pathway in mouse OA.^230^ Chitosan oligosaccharide (COS) has also been reported to inhibit synovial inflammation in vivo by activating AMPK at the early-stage OA.^231^ Administration of rapamycin, which inhibits mTOR, reduced the severity of experimental OA in mice.^232^ In addition, the safflower yellow,^233^ estrogen,^210^ puerarin,^234^ and bilobalide^235^ were reported to attenuate OA by promoting AMPK activation. They could be some novel therapeutic options for OA treatment.

Metformin is the first-line clinical drug in the treatment of type 2 diabetes.^236^ Currently, more and more studies have proved that it has not only hypoglycemic properties but also anti-aging and anti-inflammation properties.^237^ In recent years, studies of metformin in OA treatment have attracted extensive attention. Metformin, as an anti-diabetic drug, could indirectly ameliorate the negative impact of the obesity factor on OA disorder.^238^ Clinical studies have also shown that metformin had beneficial effects on long-term knee prognosis in obese patients.^239^ In addition, metformin could act as an effective activator of AMPK.^240^ Because of the important role of AMPK in pain signal transduction and metabolism of chondrocytes, studies have found that metformin also plays important roles in OA treatment by suppressing inflammation, regulating autophagy, reducing oxidative stress, and relieving pain.^227,241–244^ Inflammation can upregulate the expression of catabolic enzymes, such as ADAM, ADAMTS, and MMPs family, resulting in cartilage degeneration.^245^ Metformin acts directly or indirectly on a variety of signaling pathways, including mTOR/STAT,^246^ sirt1/NF-κB,^247^ Dicer/microRNA-34a-5p, and miR125b-5p,^248^ and HDAC5/KLF2,^249^ reduces the release of early inflammatory mediators and inhibits the inflammatory response.^244^ Autophagy is associated with maintaining cartilage homeostasis during OA development.^250^ AMPK can activate key kinases that induce autophagy.^251,252^ It has been reported that metformin treatment can enhance AMPK in a dose-dependent manner to inhibit mTORC1 and upregulate the expression of LC3, an autophagy marker, in chondrocytes.^226^ Metformin also enhanced autophagy in chondrocytes via the AMPKα2-SIRT1 signaling pathway, and the expression of autophagy-associated proteins was decreased by silencing AMPKα2 but not AMPKα1.^227^ Metformin also plays a protective role in cartilage by upregulating SIRT3, which is located in the mitochondria and regulates mitochondrial function through AMPK activation and AMPK-independent activation.^244,253^ In addition, a study showed that metformin reduced pain sensitivity in mice by upregulating AMPKα1 expression in the dorsal root ganglia (DRG).^225^ In a rat OA model induced by monosodium iodoacetate (MIA), metformin treatment decreased the expression of pain-related mediator CGRP in the DRG.^243^ In conclusion, metformin not only reduces blood glucose but also protects cartilage from degradation. Metformin is a promising therapeutic drug for OA.

### mTOR pathway

The mTOR (mechanistic target of rapamycin kinase) is a serine/threonine-protein kinase that is evolutionarily conserved and regulated by various cellular signals. It plays a key role in growth, metabolism, autophagy, and other biological processes.^254^ mTOR, which forms subunits of two protein complexes referred to as mTOR Complex 1(mTORC1) and mTOR Complex 2 (mTORC2), belongs to the phosphatidylinositol 3-kinase (PI3K)-related kinase family due to its catalytic domain being similar to lipid kinases PI3K.^255^ mTORC1 contains three key components: mTOR, Raptor (regulatory protein associated with mTOR) that facilitates substrate recruitment and subcellular localization of mTORC1, and mLST8 (mammalian lethal with Sec13 protein 8), also known as GβL that contains catalytic domain of mTORC1. The two inhibitory subunits of mTORC1 are known as PRAS40 (proline-rich Akt substrate of 40 kDa) and DEPTOR (DEP domain containing mTOR interacting protein). Similar to mTORC1, mTORC2 also contains mTOR and mLST8, as well as the Rictor instead of DEPTOR, as its three main components, and mSin1 and Proctor1/2 as its regulatory subunits.^256^ mTORC1 boosts protein synthesis by phosphorylation of S6K1 (p70S6 kinase) and 4EBP (eIF4E binding protein) and regulates protein turnover through targeting proteasome-related protein ubiquitylation and degradation.^257^ It also promotes lipid and nucleotide synthesis in fast-growing and proliferating cells and facilitates cell growth by boosting the glucose metabolic shifts from oxidative phosphorylation to glycolysis. mTORC2 regulates cell proliferation and cell survival mainly by phosphorylating and activating Akt, a key regulator of insulin/PI3K pathway.^258^ In addition, mTORC2 controls cell growth and metabolism by phosphorylating AGC protein kinase family members. mTORC1 is regulated by growth factors and response to unfavorable growth conditions, such as DNA damage, hypoxia, low ATP levels, and depletion of amino acids.^258^ However, mTORC2 is primarily regulated by insulin levels.^259^ Dysregulation of mTOR can lead to many diseases, such as cancer, obesity, diabetes, and Alzheimer’s disease.^255^

Global ablation of mTOR causes embryonic lethal in mice.^260^ mTOR is crucial for the early stage of cartilage development and growth. The expression of mTOR is significantly upregulated in human OA cartilage and in articular chondrocytes of mouse experimental OA models.^261^ Conditional deletion of mTOR in articular cartilage upregulated chondrocyte autophagy and protected mice from DMM-induced OA.^262^ However, genetic evidence of the distinctive roles of mTORC1 and mTORC2 in articular cartilage and OA development is still missing. Activation of mTORC1 by cartilage-specific inactivation of Tsc1 (tuberous sclerosis complex 1), a mTORC1 upstream inhibitor, stimulated articular chondrocyte differentiation to initiate OA by downregulating parathyroid hormone (PTH)/PTH-related protein (PTHrP) receptor and FGFR3.^263^ The deletion of PPARγ (peroxisome proliferator-activated receptor-γ) in mouse chondrocytes increased mTOR expression, resulting in the overproduction of OA inflammatory and catabolic factors, which caused chondrocyte apoptosis, cartilage degradation, and accelerated OA progression.^264^ In contrast, PPARγ-mTOR double KO mice displayed delayed OA acceleration caused by the PPARγ loss [10].

Inhibition of mTOR expression in chondrocytes enhanced the expression of AMPK and the autophagy gene LC3 and ULK1.^232^ The interaction between ULK1/AMPK and the mTOR signaling pathways is a critical component in the regulation of autophagy (Fig. 5). Activation of mTORC1 decreased the phosphorylation of ULK-1, thus inhibiting the activation of ULK1, leading to the suppression of the autophagy.^265^ Pharmacological inhibition of mTOR by rapamycin enhanced cartilage matrix production and the content of collagen type II and decreased the expression of cartilage matrix-degrading proteins, including ADAMTS5 and IL-1β in articular cartilage.^232^

Abnormal mechanical loadings play an important role in the occurrence and progression of the OA.^266^ Abnormal mechanical stress caused degradation of articular cartilage by triggering the mTORC1 signaling, which modulated the apoptosis and autophagy of chondrocytes in TMJ (temporomandibular joint) both in vitro and in vivo.^267^ Subchondral bone has been reported to play a regulatory role in OA development. The mTORC1 was reported to be activated in the osterix-positive osteoblast in the subchondral bone of human and mouse OA. Eliminating TSC1 (tuberous sclerosis 1), a mTORC1 inhibitor, to constitutively activate mTORC1 in pre-osteoblasts stimulated osteosclerosis and CXCL12 secretion, thus exacerbating OA. Meanwhile, suppressing mTORC1 by deleting the mTORC1 component, Raptor, inhibited the formation of subchondral bone and degeneration of the cartilage of anterior cruciate ligament transection (ACLT) induced OA in mice.^268^

mTOR is also associated with the level of synovial inflammation.^269^ Rapamycin, a specific inhibitor of mTORC1, reduced ADAMTS5 and IL-1β levels in the synovitis of the knee joint in OA mice.^232^ microRNAs (miRNAs), as one of the epigenetic mechanisms that regulate gene expression,^270^ play a crucial role in OA. The miR-7, miR-27a, and miR-218-5p were reported to inhibit the activity of the PI3K/Akt/mTOR signaling pathway, thus enhancing autophagy and inhibiting the apoptosis of chondrocytes.^271–273^ In addition, exosomes derived from MSCs in the sub-patellar fat pad protected mice from DMM-induced OA partially by promoting autophagy through suppression of mTOR by miR-100-5p.^274^

Inhibition of the mTOR pathway by using rapamycin activated autophagy in the knee joint of mice and thus reduced the severity of experimental OA.^232^ Similarly, the hydroethanolic extract of Butea monosperma (BME) activated autophagy and inhibited IL-1β-induced MMP-3 and IL-6 expression by inhibiting the mTOR.^275^ Intra-articular injection of mTOR inhibitors, such as rapamycin, in the early stage of OA, is a promising therapeutic strategy for OA treatment.

### HIFs pathway

Mammalian cells respond to low oxygen tension microenvironments by activating hypoxia-inducible factors (HIFs) to help the cell survival.^276^ Functional HIF proteins are heterodimers consisting of an α-subunit (HIF-α), which is encoded by *HIF1α*, *HIF2α*, or *HIF3α* three genes, and a β-subunit (HIF-β), also referred to as the ARNT (aryl hydrocarbon receptor nuclear translocator).^277^ When the oxygen tension is normal, HIF-1α is hydroxylated by three HIF-specific prolyl hydroxylases, PHD1, PHD2, and PHD3. The hydroxylated HIF-α protein is tagged by the pVHL (von Hippel-Lindau) factors with polyubiquitin, and subsequently targeted for degradation.^277^ When the cells are under hypoxic conditions, the activity of PHDs is inhibited, leading to impaired hydroxylation and accumulation of HIF-α proteins in the cytoplasm.^277^ The accumulated HIF-α protein is then translocated to the nucleus. It binds with HIF-β to function as a transcriptional factor to regulate hundreds of genes, including erythropoietin (EPO), glucose transporter-1 (GLUT1), and vascular endothelial growth factor A (VEGFA).^277^

HIF-1α is a crucial survival factor for hypoxic chondrocytes in vivo.^278^ It has been recently demonstrated that HIF-1α promotes hypoxic chondrocyte survival by suppressing mitochondrial respiration in the fetal growth plate in vivo.^279–281^ In contrast, the function of HIF-2α in endochondral bone development has shown controversial results. HIF-2α heterozygous global knockout mice showed impaired endochondral ossification by decreasing the Col10a1 expressing and suppressing hypertrophic differentiation and matrix degradation.^282^ On the other hand, it is reported that homozygous loss of HIF-2α in limb bud mesenchyme cells achieved by using conditional knockout strategy led to only a modest delay of endochondral bone development in the prenatal stage without affecting Col10a1 expression and accumulation. The delayed bone development phenotype was transient and not detectable in the postnatal stage in mice.^283^ The mechanism leading to this discrepancy requires further investigation.

**Fig. 6:**
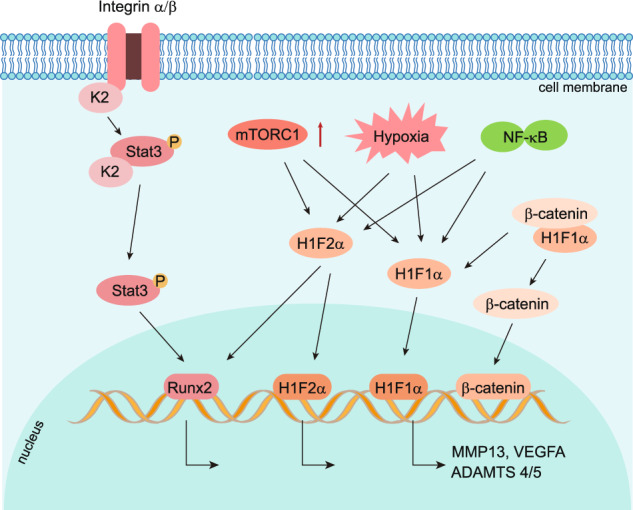
Role of focal adhesion and HIFs pathway in OA chondrocytes. Focal adhesion protein Kindlin-2(K2), which is co-localized with integrin α on the cell membrane, is highly expressed in adult articular chondrocytes. In OA chondrocytes, loss of Kindlin-2 promotes chondrocyte hypertrophy and catabolism by activating the phosphorylation of Stat3 and upregulating transcription factor Runx2. HIF-1α and HIF-2α are both activated by hypoxia. HIF-1α inhibits Wnt signaling by blocking β-catenin translocation to the nucleus and downregulates MMP13 expression in healthy articular cartilage in mice. HIF-1α λ is upregulated in OA chondrocytes and induces abnormal blood vessel invasion by upregulating VEGFA. HIF-2α directly induces the expression of OA risk factor, Runx2, and catabolic genes encoding MMP1, MMP3, MMP13, ADAMTS4, and NOS2, to promote cartilage destruction in OA chondrocytes. Besides hypoxia, the activation of the NF-κB pathway and the upregulation of mTORC1 induce the overexpression and excessive accumulation of HIFs protein in articular chondrocytes

Articular cartilage is an avascular tissue and has access to minimal oxygen.^284,285^ Therefore, articular chondrocytes are under hypoxia in physiological conditions, and accumulated HIF-1α and HIF-2α proteins were detected both in mice and human.^286,287^ The number of HIF-1α-positive articular chondrocytes was decreased at the early stages of post-traumatic OA mice models.^285,288^ HIF-1α protein inhibited Wnt signaling by blocking TCF4-β-catenin interaction and down-regulated MMP13 expression to protect articular cartilage loss that induces OA phenotype in mice.^285,288^ It suggests that HIF-1α acts as a protector of articular cartilage integrity in physiological conditions (Fig. 6). Several reports showed that HIF-1α and its target genes were expressed in normal human knee joint articular cartilage and were significantly increased in the cartilage and synovial fluid of joints of OA patients.^286,289^ In addition, HIF-1α-positive chondrocytes were increased with the severity of OA,^286,289^ and miRNA-411 were reported to target HIF-1α in regulating chondrocyte autophagy in OA.^290,291^ Then, HIF-1α was demonstrated to act as a protector to alleviate apoptosis and cell death in OA by promoting mitophagy in OA cartilage,^292^ which supports the notion that HIF-1α serves as a promising strategy for OA treatment. Activation of HIF signaling by conditional knocking out VHL in articular chondrocytes led to cartilage degradation in aged mice but did not affect the gross appearance of articular cartilage in young mice.^293^ The HIF-1α protein level was similar in VHL knockout articular cartilage compared to that in control mice, while the HIF2α protein was significantly increased.^293^ HIF-1α is downregulated in the articular chondrocytes at the early stage of OA but significantly upregulated at the late stage of OA in humans and mice. However, the effect of overexpression of HIF-1α in articular cartilage in transgenic mouse models remains to be determined to further identify the role of HIF-1α in OA development. The expression of HIF-2α showed a marked increase in OA cartilage compared to that on normal cartilage in mice and in humans, and HIF-2α-heterozygous deficient mice showed resistance to OA development.^282,287^ Overexpression of HIF-2α triggered the destruction of articular cartilage in mice and rabbits.^287^ In contrast to HIF-1α, HIF-2α was reported as a risk factor that directly induces the expression of OA risk factor Runx2 and catabolic genes, including MMP1, MMP3, MMP13, ADAMTS4, and NOS2, to promote cartilage destruction.^287^ There are few studies about the function of HIF-3α in cartilage and OA development. Besides hypoxia, the activation of the NF-κB pathway induced by cellular inflammation and the upregulation of mTORC1 could also induce the overexpression and excessive accumulation of HIFs protein in articular chondrocytes.^294,295^

Two new PHD2 inhibitors have been synthesized, known as TM6008 and TM 6089, to selectively inhibit PHD2, but not PHD1 and PHD3, to activate the HIF pathway.^296^ Other PHDs inhibitors, such as FK506-binding protein 38 (FKBP38) and desferrioxamine (DFO), have also been tested in animal models.^296^ However, the strategy of inhibiting PHD2 as a therapeutic target for OA is a double-edged sword since both HIF-1α and HIF-2α will be activated after the inhibition of PHD2. The benefit of accumulating HIF-1α could be neutralized by also activating the OA risk factor HIF-2α. Therefore, the specific HIF-2α inhibitor is an alternative promising therapeutic strategy. Resveratrol and YC-1 (3-(5’-hydroxymethyl-2’-furyl)-1-benzylindazole) were reported to prevent OA progression in vivo by suppression of HIF-2α indirectly in mice.^297,298^ Given that HIF-1α and HIF-2α have the opposite function in OA progression, a combination treatment of PHD2 inhibitor and specific HIF-2α inhibitor could be a potential strategy.

### Focal adhesion pathway

Focal adhesions (FAs) are complex macromolecular assemblies that form dynamic mechanical links between intracellular actin bundles and the ECM to control a series of fundamental cellular processes, such as cell adhesion, migration, and signal transduction.^299–303^ Integrins are heterodimer transmembrane receptors consisting of one α subunit and one β subunit, which facilitate cell-cell and cell-ECM adhesion. After integrins anchoring with ECM proteins, many structural and signaling proteins, such as talin, kindlin-2, vinculin, paxillin, pinch1/2, zyxin, α-actinin, focal adhesion kinase (FAK), and phosphotyrosine proteins, are recruited to the cell-ECM contact sites to form FAs.^299^ During the last decade, cumulating evidence has pointed out that the FAs signaling plays a central role in controlling organogenesis, metabolism, and homeostasis.^179,300–311^ Genetic ablation of FAs-related genes in mesenchymal stem cells leads to severe chondrodysplasia, neonatal lethality, and skeletal abnormalities.^303,311,312^ More importantly, critical molecules of FAs, such as kindlin-2 and integrins, have been implicated in the pathogenesis of OA, considering their essential functions of mediating mechanotransduction, articular cartilage homeostasis, synovial activation, and subchondral bone remodeling.^313–315^ For instance, Lian and coworkers have reported that ECM protein type II collagen promoted the interaction between integrin β1 and Smad1, thereby suppressing articular chondrocyte hypertrophy during OA development.^316^ Moreover, changes in subchondral bone architecture altered the distribution of mechanical stress on articular cartilage and induced a talin-dependent cytoskeletal reorganization and the consequent increase of cell contractile forces and cell stiffness of articular chondrocytes, leading to αV integrin-mediated TGFβ activation.^317^ The latter further disrupted the cartilage homeostasis by enhancing the catabolic activity of articular chondrocytes, leading to OA progression.^317,318^ An increased expression of αVβ6 integrins was observed in OA synovial tissues, which also mediated TGF-β activation through their interaction with a protein fragment of vitronectin (amino acid 381–397).^319^ Furthermore, dysregulation of integrin αVβ3 and the integrin-associated receptor CD47 signaling promoted synovial inflammation, cartilage degradation, and OA progression.^320^ The roles of integrins in OA development and progression have been recently reviewed by Jin and colleagues.^313^

A recent study by our group has revealed a crucial role of key FA protein kindlin-2 in maintaining articular cartilage homeostasis in adult mice.^308^ We found that kindlin-2 is highly expressed in adult articular chondrocytes. Genetic deletion of kindlin-2 in these cells caused multiple OA-like phenotypes, including spontaneous cartilage degradation and synovial inflammation, subchondral bone sclerosis, and hyperalgesia. In in vitro and in vivo experiments, kindlin-2 deficiency promoted chondrocyte hypertrophy and catabolism by activating the Stat3-Runx2 pathway in articular chondrocytes (Fig. 6). Mechanistically, we found that kindlin-2 was robustly expressed in the mitochondria of articular chondrocytes, and loss of kindlin-2 resulted in mitochondrial dysfunction and excessive production of ROS. Moreover, we demonstrated that kindlin-2 is bound to the Stat3 protein to inhibit the ROS-mediated activation of the Stat3-Runx2 pathway in chondrocytes. Intraarticular injection of kindlin-2-expressing adeno-associated virus attenuated aging- and instability-induced OA damages in mice. Similarly, pharmacological inhibition of FAK decelerated articular cartilage degeneration and subchondral bone deterioration in a surgically induced rat OA model.^321^ Pharmacological inhibition of FAK relieved the degradation of articular cartilage of the mandibular condyle under mechanical loading by inhibiting the expression of pro-inflammatory cytokines and ECM-degrading enzymes.^322^ Miyauchi and coworkers showed that genetic deletion of the Hic-5 (hydrogen peroxide-inducible clone-5), a focal adhesion mechanosensitive adapter, alleviated the progression of OA through regulation of chondrocyte catabolism induced by mechanical stress.^323^

Taken together, current evidence suggests that the FAs signaling plays a critical role in the pathogenesis of OA. Further studies are needed to explore the molecular mechanisms underlying how alterations in the FAs signaling contribute to the initiation, development, and progression of OA.

### TGFβ and BMP signaling pathways

**Fig. 7:**
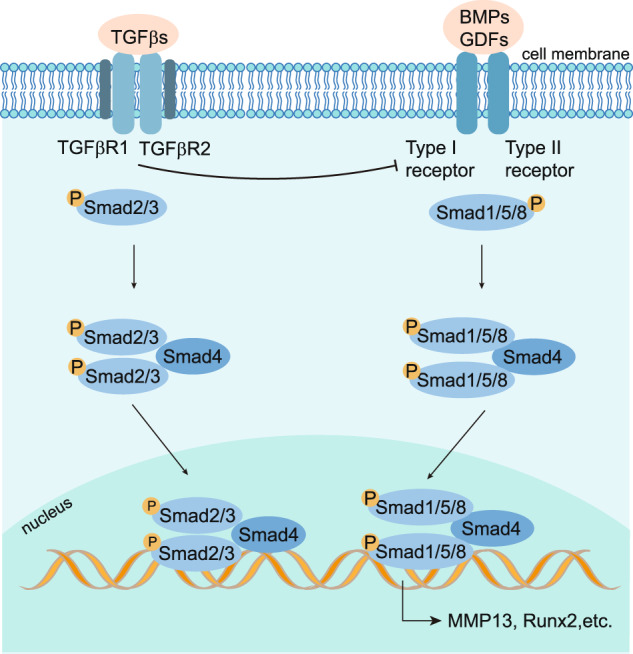
Schematic representation of TGFβ/BMP signaling pathway in OA chondrocytes. TGF-β/BMP family ligands bind to type I and type II receptors on the cell surface. TGF-βs bind TGFβRI and TGFβRII, and BMPs bind BMPRI and BMPRII. TGFβRIII is a coreceptor that facilitates interaction with TβRI and TβRII. TGF-βs induce the phosphorylation of Smad2/3, and BMPs mediate Smad1/5/8 phosphorylation. By forming complexes with Smad4, phosphorylated Smad2/3 and Smad1/5/8 translocate into the nucleus to regulate target gene expression

Growth factor transforming growth factor β (TGFβ) superfamily consists of BMPs (bone morphogenetic proteins), TGFβs, growth/differentiation factors (GDFs), and other factors.^324,325^ TGFβ/BMP signaling is transduced by heteromeric transmembrane receptor complexes composed of type I and type II serine/threonine kinase receptor dimers. Seven Type I and five Type II receptors have so far been identified. Several type III receptors have been identified to stabilize the receptor complexes and facilitate the interaction between the ligand and receptor. TGFβ/BMP signaling activates both SMAD-dependent signaling (referred to as the canonical signaling pathway) and SMAD-independent signaling in cells. In the canonical pathway, transmembrane receptors are activated by specific ligands binding, followed by phosphorylation of R-SMAD (receptor-regulated Smads) proteins. Phosphorylated R-SMAD is then translocated to the nucleus and triggers transcriptions of downstream target genes. There are two sets of R-Smad complexes: one set consists of Smad1, Smad5, and Smad8 (also known as Smad9), and the other set is composed of Smad2 and Smad3.^326^ After receptor activation, these phosphorylated R-Smads are then combined with co-Mediator Smad (co-Smad) Smad4 to form a complex and transported to the nucleus to perform the function of transcriptional factor (Fig. 7). TGFβ/BMP can also activate SMAD-independent signaling pathways, including TAK1(TGFβ−activated kinase 1) and ERK1/2 (extracellular signal-regulated kinase 1 and 2).^327,328^

A series of studies have shown that TGFβ/BMPs are an indispensable signaling pathway for regulating cartilage homeostasis both through canonical and non-canonical pathways.^329–331^ TGFβ/BMPs signaling protects articular cartilage and participates in chondrocyte hypertrophy and ECM degradation.^332^ BMP2, BMP4, BMP6, and BMP7 are expressed in articular cartilage and induce the production and proliferation of ECM proteins, such as proteoglycan and collagen type II.^333–338^ BMP2 knockout mice formed un-mature knee joints and had dysfunctional meniscus and articular cartilage defects, leading to progressive OA with age.^339^ Although BMP7 had similar effects to BMP2, BMP7-Smad1/5 signaling was not associated with chondrocyte hypertrophy.^340,341^ BMP7 knockout mice exhibited increased proteoglycan loss and Mmp13 expression.^342^ Meanwhile, the signaling crosstalk between the BMP signaling pathway and the Wnt/β-catenin signaling pathway plays an important role in regulating cartilage homeostasis.^343^ Loss of TGFβR1 in mouse growth plate cartilage increased basal BMP activity, suggesting that TGFβR1inhibits BMP signaling in the development of the growth plate.^344^ However, the role of TGFβR1 and TGFβR2 in the homeostasis of articular cartilage is still unknown. How the non-canonical BMP pathway regulates the homeostasis of the articular cartilage and contributes to the OA onset and development remains to be determined.

The BMP signaling pathway is abnormal and unstable in OA patients.^345^ The expression of BMP-2 and BMP-4 in chondrocytes of articular cartilage of OA patients was significantly upregulated in the early stage and increased with OA degree. BMP-4 promoted the pathological remodeling of the osteochondral junction.^346^ OA significantly promoted BMP2 and MMP-13 expression in the subchondral bone of the experimental OA rat model. This increase was inhibited by intra-articular injection of noggin protein (a BMP2 inhibitor). Meanwhile, Noggin protein dramatically attenuated OA disease progression in early OA rat model.^347^ Moderate exercises increased the expression of BMP signaling pathway-associated proteins, such as BMP2, BMP6 and BMP receptor 2, and pSmad-5, thus inhibiting cartilage degeneration and attenuating the OA phenotype.^348^

More and more studies focus on the potential therapeutic targets of OA. miR-181a is critical for crosstalk between the BMP and Wnt/β-catenin signaling pathways and may become a target for OA therapy.^349^ BMP7-derived peptides ameliorated OA chondrocyte phenotype in vitro and attenuated cartilage degeneration in vivo.^350^ The clinical trial phase II of the intra-articular injection of BMP-7 for the treatment of knee OA patients was completed (NCT01111045) in 2011. BMP receptor type I (BMPRI) mimetic peptide CK2.1 promoted articular cartilage repair and inhibited chondrocyte hypertrophy through intra-articular injection.^351^ Several studies showed that BMP-2 and BMP-4 effectively decreased cartilage degeneration in combination with tissue engineering materials. Recombinant human BMP-2 promoted cartilage regeneration in injured cartilage in vitro and was evaluated in clinical trials (NCT00243295). Tissue engineering materials, such as porous Hydroxyapatite Collagen (HAp/Col), Conically GRADED scaffold of chitosan-hax Hydrogel/Poly (L-Lactide-co-Glycolide) (PLGA), and POLY caprolactone (PCL) scaffolds, combined with BMP-2 treatment greatly promoted cartilage regeneration and repaired the joint cartilage.^352–354^ BMP-3 inhibited cartilage regeneration in both partially and completely defective rabbit joint injury models by inducing ECM degradation, inhibited the expression of BMP-2 and BMP-4, and interfered with chondrocyte survival on the articular surface.^355^ The intra-articular injection of BMP-4 combined with muscle-derived stem cells (MDSCs) could efficiently repair articular cartilage damage in the experimental OA rat model.^356^ Regular intra-articular injections of BMP7 alleviated the damage to articular cartilage caused by strenuous running.^357^ The use of BMP6 combined with BMP2 or TGFB3 induced enhanced chondrogenesis of stem cells in vitro.^358,359^ The combined use of an osteogenic nanoparticulate mineralized glycosaminoglycan scaffold (MC-GAG) and BMP-9 promoted chondrogenic differentiation of primary human mesenchymal stem cells (hMSCs) by inducing increased expression of collagen II, aggrecan and cartilage oligomeric protein.^360^

### FGF signaling pathways

The fibroblast growth factor (FGF) family is present in a wide range of animal species, including nematodes, zebrafish, mice, and humans.^361^ FGF signaling is involved in a variety of physiological processes, such as cell proliferation, migration, and differentiation.^362^ There are 18 FGFs in mammals, and they are FGFs1-10 and FGFs16-23. FGFs19, 21, and 23 are hormone-like FGFs that work in an endocrine fashion, and the other FGF members work in a paracrine fashion. FGFs ligands fulfill their functions through binding and activating FGF receptor (FGFR).^362^ The mammalian FGFR family consists of four members, FGFR1, FGFR2, FGFR3, and FGFR4. FGF signaling involves multiple downstream signaling pathways, including the RAS/MAPK, PI3K/AKT, and PLCγ pathways. In addition, FGF signaling can activate the STAT1/p21 pathway.^363^

FGF signaling plays a critical role in bone and cartilage development.^363^ FGFs/FGFRs function at various stages of bone and cartilage development from limb bud formation to long bone growth and maturation.^363^ FGFR1 and FGFR3 are the predominantly expressed FGF receptors in cartilage. FGFR2 expression is restricted to the pre-cartilage condensate zone, and FGFR2 can serve as an early marker of chondrocytes.^364^ During growth plate formation, FGFR1 is expressed in pre-hypertrophic and hypertrophic regions, and FGFR2 is observed in quiescent regions.^365^ FGFR3 was detected in the center of the mesenchymal condensation and all growth plate chondrocytes.^364,365^ At the same time, FGFs 1, 2, 5, 8, 9, 16–19, 21, and 23 are expressed in the growth plate chondrocytes, and FGFs 1, 2, 6, 7, 9, 18, 21, and 22 are expressed in the perichondrium.^366^

Previous studies have shown that FGFs 1, 2, 7, 8, 9,18, and 23 are the major ones associated with the pathogenesis of OA (Table 2), but they have diverse functions in the development of the joint disorder.^367^ The secretion and expression of FGF1 were significantly increased in the synovium in the late stages of the OA. FGF1 was reported to suppress ECM synthesis of the human articular chondrocytes and inhibit the expression of CCN2 (cellular communication network factor 2), which is an important factor that promotes the regeneration of damaged cartilage.^368^ FGF2 was reported to bind to perlecan, a heparan sulfate proteoglycan in the ECM, and functioned as a mechanosensor in articular cartilage.^369^ FGF2 binds to FGFR1 to upregulate the expression of MMP1 and MMP13 promoting matrix degradation through neuro-endocrine pathways in adult articular chondrocytes.^370,371^ FGF8 expression is upregulated in damaged synovium in a rabbit model of OA, and FGF8 was shown to enhance the production of proteases and prostaglandin E2 in inflamed synovial cells, thereby promoting cartilage degradation.^372^ The expression of FGF9 was downregulated in the human OA cartilage.^373^ Treatment of exogenous FGF9 attenuated cartilage degeneration but exacerbated the osteophyte formation in a mouse OA model.^373^ The function of FGF9 in articular cartilage remains to be defined. FGF18 was highly expressed in the superficial zone of articular cartilage and stimulated the expression and accumulation of type II collagen in articular chondrocytes to protect articular cartilage against degeneration.^374–376^ In addition, the FGF signaling pathway also plays an important role in synovitis. The synthesis and secretion of FGF1 were significantly increased in the synovial fibroblast in OA.^377^ FGFR2, which is one of the cognate receptors of FGF1, was upregulated in the synovial membrane in OA patients.^377^ FGF2 is a potent agent to promote the proliferation and chondrogenesis of synovial-derived stem cells.^378^ The expression of FGF8 was significantly upregulated in hyperplastic synovial cells and fibroblasts in the rabbit OA model.^372^ Deletion of FGFR1 in adult mouse articular chondrocytes inhibited the progression of articular cartilage degeneration, which was associated with MMP13 downregulation and FGFR3 upregulation.^379^ Zhou S et al. showed that deletion of FGFR3 in articular chondrocytes in mice resulted in OA-like defects in the temporomandibular joint, which were associated with upregulation of RUNX2 and Indian hedgehog (IHH).^380^ This suggests that targeting FGF may have a potential strategy for OA treatment. The clinical trials phase II of the only FGF-targeting drug for OA, recombinant human FGF18 (sprifermin), was finished in 2020 (NCT01919164). Intra-articular administration of 100 μg Sprifermin every 6 or 12 months significantly increased the thickness of the femorotibial joint cartilage after two years of treatment with no marked side effects.^381^

**Table 2:** The expression changes of key proteins of each signaling pathway in the synovial joint

Key protein	Expression location in human	Expression in human OA	Expression in animal OA	References
FGF1	Synovial and articular chondrocytes	Upregulation	Upregulation in MIA-induced KOA in rats	PMID: 21835001
FGF2	Articular chondrocytes	Unknown	low-molecular-weight FGF2 isoform upregulates in spontaneous OA in mice; while loss of high-molecular-weight FGF2 isoforms downregulates in spontaneous OA in mice	PMID: 17960584
FGF7	OA meniscal cells	Upregulation	Upregulation in human OA meniscal cells	PMID: 20109188
FGF8	Unknown	Unknown	Produced by injured synovium in DMM-induced OA in rabbit	PMID: 18699993
FGF9	Articular cartilage	Downregulation	Downregulation in DMM-induced OA in mice;	PMID: 22841919
FGF18	Articular cartilage	Downregulation	Downregulation in DMM-induced OA in mice;	PMID: 24577103
FGF23	OA chondrocytes	Upregulation	Upregulation in posttraumatic OA in rats	PMID: 29718273
FGFR1	Articular cartilage	Upregulation	Upregulation in spontaneous and DMM-induced OA in mice;	PMID: 21835001
FGFR2	Articular cartilage	Upregulation		PMID: 26400350
FGFR3	Articular cartilage; OA synovial tissue	Downregulation	Downregulation in spontaneous and DMM-induced OA in mice;	PMID: 27041063

*OA* osteoarthritis, *KOA* knee osteoarthritis, *FRZB* frizzled-related protein, *DMM* destabilization of the medial meniscus, *MIA* monosodium iodoacetate, *ACLT* anterior cruciate ligament transection, *STR/ort mice* a well-recognized mouse model which develops spontaneous osteoarthritis very similar to the human disease

### Runx2

**Fig. 8:**
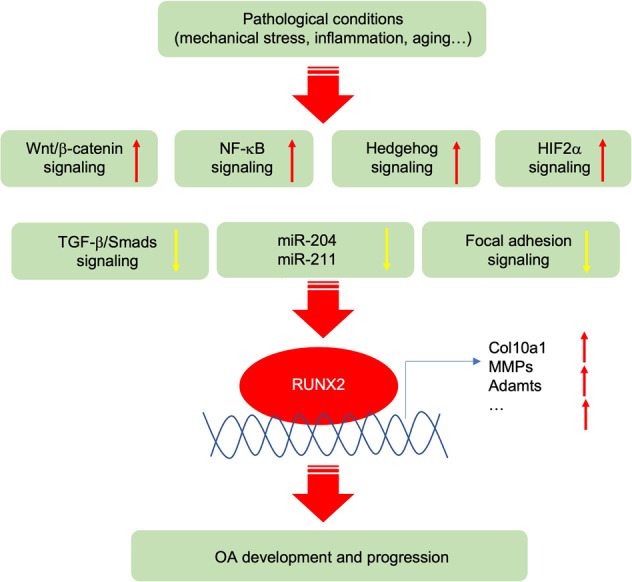
Runx2 in OA pathogenesis. A diagram showing the upstream and downstream of Runx2 in OA pathogenesis. Runt-related transcription factor 2 (Runx2) is upregulated by activation of Wnt/β-catenin, NF-κB, Hedgehog, HIF-2α signaling, and by suppression of TGF-β/Smads and focal adhesion signaling in OA chondrocytes. miR-204 and miR-211 inhibit the expression of Runx2 protein in articular chondrocytes in vivo. Red arrow: upregulated expression. Yellow arrow: downregulated expression

Runt-related transcription factor 2 (Runx2) is a runt domain-containing transcription factor that binds to DNA as a monomer or, with higher affinity, as a part of a heterodimeric complex.^382^ During skeletal development, Runx2 is predominantly expressed by pre-hypertrophic and hypertrophic chondrocytes and osteoblast-linage cells and functions as a potent inducer of hypertrophic chondrocyte differentiation as well as osteoblastic differentiation.^383–389^ Homozygous deficiency of Runx2 in mice caused neonatal lethality with a complete lack of skeletal ossification, whereas heterozygous Runx2 deficiency led to cleidocranial dysplasia, characterized by defective clavicle and skull formation.^390,391^ Although the expression of Runx2 is essential for skeletal development and bone remodeling, numerous studies have recently suggested that Runx2 also acts as a central pathological factor in OA initiation, development, and progression (Fig. 8). This notion is supported by: (1) elevated expression of Runx2 is a standard feature found in OA cartilage samples from humans and animals^130,308^; (2) chondrocyte-specific overexpression of Runx2 hastened the progression of posttraumatic OA in adult mice^392^; (3) genetic deletion of Runx2 in articular chondrocytes attenuated the severity of surgically-induced OA lesions in adult mice, and (4) Runx2 directly regulated the mRNA and protein expressions of chondrocyte hypertrophic marker Col10a1 and catabolic enzymes, such as Mmp13 and Adamts5.^393,394^ In addition, Runx2 can act as a downstream factor of multiple key signaling pathways, including Wnt/β-catenin, TGF-β/Smad, Indian hedgehog (Ihh), hypoxia-induced factor (HIF), and focal adhesion signaling pathways, to initiate the processes of hypertrophic chondrocyte differentiation and articular cartilage catabolism upon pathological stimulations.^130^ Despite a well-established role in articular cartilage, Runx2 has also been linked to pathological changes in various joint tissues, such as the meniscus, synovium, and subchondral bone.^130^ Notably, the involvement of Runx2 in OA pathogenesis has been extensively reviewed by Chen et al. and Zhao et al..^130,395^ Although recent studies have demonstrated a critical role of Runx2 in OA pathogenesis and highlighted it as a potential therapeutic target,^130,308,393^ currently, there are no Runx2-targeting drugs or therapies have been developed for the treatment of OA. Considering the essential roles of Runx2 in other organs and tissues,^396–398^ the recently developed chondrocytes-targeted delivery systems may serve as useful approaches for the specific delivery of Runx2 siRNAs or inhibitors, such as CADD552.^399–402^ Moreover, the CRISPR/Cas9 system has demonstrated high efficiency in gene editing in multiple joint tissues.^403^ Intraarticular injections of adeno-associated virus (AAV) expressing Runx2-targeting miRNA sequences significantly downregulated the expression of Runx2 and decelerated OA progression in mice.^131^ Nonetheless, new Runx2-targeting drugs still need to be designed, and further studies should be conducted to evaluate their therapeutic potential for OA treatment in animal models and humans.

Alterations in the expression patterns of miRNAs were observed in serum and cartilage samples from OA patients, suggesting that miRNAs can serve as biomarkers for OA diagnosis and classification.^404,405^ In vitro and in vivo studies have demonstrated that miRNAs can regulate articular chondrocyte differentiation, matrix catabolism, inflammatory responses, autophagy, and chondrocyte apoptosis.^406–411^ miRNAs also control cellular signal transductions by mediating the expression levels of key transcriptional factors, such as Runx2. For instance, Huang and coworkers reported that two homologous microRNAs, i.e., miR-204 and miR-211, exerted multiple crucial functions in maintaining the homeostasis of articular cartilages via targeting the Runx2 expression.^131^ Genetic deletion of miR-204/211, either globally or selectively in mesenchymal progenitor cells, resulted in striking OA-like phenotypes, including deterioration of articular cartilage, osteophyte formation, subchondral bone sclerosis, and synovial hyperplasia.^131^ Notably, miRNAs participate in intracellular signal transduction and can also be utilized as mediators in miRNA-based intercellular communication. A recent study has revealed that miRNAs derived from subchondral osteoclasts can transfer to articular chondrocytes via exosomes, contributing to cartilage degeneration, osteochondral vessel invasion, and sensory innervation during OA development.^412^ These findings suggest that miRNAs play an important role in cartilage homeostasis and OA development.

### Matrix metalloproteinases

Matrix metalloproteinases (MMPs) are a family of zinc-dependent proteolytic enzymes that cleave ECM proteins. One type of MMPs is called collagenase, including MMPs1, 8, and 13, which function as collagen-degrading enzymes and contribute to the catabolic turnover of articular cartilage. These MMPs, especially the MMP13, are closely associated with the ECM destruction of articular cartilage in OA. One substrate of MMP13 is type II collagen which is one of the most abundant structural components in articular cartilage. Compared to wild-type mice, MMP13 global knockout mice exhibited reduced articular cartilage degradation in DMM-induced mouse OA models, and constitutive overexpression of MMP13 in cartilage caused OA-like phenotypes in mice.^413^ Consistent with this, conditional knockout MMP13 in chondrocytes of adult mice decelerated progression at the early and middle stage of OA (8, 12, and 16 weeks post-meniscal-ligamentous injury, MLI, surgery).^414^ As the primary collagenase in OA pathogenesis, MMP13 was slightly upregulated in articular cartilage and significantly in the synovium at the early-stage OA, specifically at 1- and 3 weeks post-surgery of the rat medial meniscus transection (MMT) OA model.^415^ MMP2, MMP3, and MMP14 are significantly upregulated at one day, one week, and six weeks time points in articular cartilage of DMM-induced mouse OA models.^416^ Inhibiting MMP13 or other MMPs at the early- stage of OA could be a potential therapeutic strategy to prevent cartilage destruction and the OA initiation process. Several natural compounds, such as curcumin and resveratrol, were reported to suppress MMP13 expression. Some zinc-binding inhibitors specific to MMP-13, such as N-O-isopropyl sulfonamide-based hydroxamates, were shown to be effective in an in vitro cartilage degradation model.^417^ Additionally, several highly selective non-zinc binding MMP-13 inhibitors, including pyrimidines, biaryls, and diphenylethers, have been reported recently.^418^ The monoclonal antibody of MMP-13, which is highly specific over other MMPs, is a potential therapeutic approach,^419^ despite its difficulty penetrating the cartilage. PF152 has been demonstrated as an effective MMP-13 inhibitor that can decrease human cartilage degradation ex vivo in a dosage-dependent manner.^420^ MMPs inhibitor PG-530742 (PG-116800) completed its phase II clinical trials ten years ago. However, the results showed that its side effects make it unsuitable for clinical use in humans.^421^

### ADAMTS and ADAMs

A Disintegrin and Metalloproteinase with Thrombospondin Motif (ADAMTS) are proteins that mediate the degradation of the ECM of chondrocytes leading to the distraction of cartilage integrity. ADAMTS family contains 19 secreted zinc metalloproteinases protein members. ADAMTS4/5 are the essential proteases expressed in human and animal OA cartilage tissues that play important roles in the degradation of aggrecan, the major component of cartilage ECM. Expression of ADAMTS4/5 was significantly elevated in articular chondrocytes even at the early-stage OA. ADAMTS5 deletion protected joints from cartilage destruction in surgically induced mouse OA model.^422,423^ The phase II clinical trials of the film-coated tablets GLPG1972/S201086, which is developed as an ADAMTS5 inhibitor for oral use for OA treatment finished in July 2021 (NCT03595618).In addition, anti-ADAMTS-5 Nanobody (M6495) for OA treatment finished the phase I clinical trials (NCT03224702) in May 2019. ADAMTS1, 2, 3, 7, 12, 14, and 16 were also upregulated at the early-stage OA. ADAMTS8 was expressed in articular cartilage, and its expression level was not changed in the articular cartilage of mouse OA models but increased in the human OA synovium.^424,425^ There are controversial reports about how ADAMTS15 expression level changes in OA cartilage. A Disintegrin and Metalloproteinases (ADAMS) are proteins responsible for removing membrane-located receptors. Out of 34 known ADAMS proteins, ADAM-8, -9, -10, -12, and -28 were reported to be overexpressed at the early stage of the OA articular cartilage.^425^ Expression of ADAM-17 was not markedly altered in human OA versus normal articular cartilages.^426^ ADAM-19 and -23 were upregulated in the late stage of the OA cartilage.^427^

### Lubricin/proteoglycan-4 (PRG4)

Healthy articular cartilage is coated on the surface by lubricin molecules, which provide lubrication boundary and low friction of the cartilage surface. Lubricin encoded by the *Prg4* (proteoglycan 4) gene is a secreted proteoglycan protein that is predominantly expressed by chondrocytes in the superficial zone of articular cartilage and synovial fibroblasts.^428,429^ Patients with a genetic mutation of the *PRG4* gene have the CACP (camptodactyly-arthropathy-coxa vara-pericarditis) syndrome, characterized by childhood onset non-inflammatory joint disorder.^430,431^ Prg4 global knockout mice develop aging-related joint disorders with loss of chondrocytes in the superficial zone of articular cartilage and synovial cell hyperplasia.^432^ Running and fluid flow shear stress could promote the expression of Prg4 in the superficial zone chondrocytes in vivo and in vitro,^433^ suggesting the regulation of Prg4 expression can be partially controlled by mechanical forces. As a protective factor for joints, Prg4 is expressed by embryonic joint progenitors. Prg4 positive articular chondrocytes located on the surface of joint cartilage in adult mice have been demonstrated to be the progenitor for deeper layers of the mature articular cartilage.^128,434^ Exogenous recombinant human (rh) PRG4E was reported to promote ear wound closure and tissue regeneration by increasing VEGF expression and blood flow through a TLR (Toll-like receptor)-dependent mechanism in mice.^435^ Full-length recombinant human PRG4 (rhPRG4) produced by CHO-M cells and native human PRG4 (nhPRG4) purified from culture supernatants of human fibroblast-like synoviocytes from OA patients were also shown to bind to TLR2 and TLR4, mediating an anti-inflammatory factor.^436^ However, whether overexpressing Prg4 in the synovial joint can facilitate articular cartilage regeneration or prevent OA development remains to be determined. Lubricin was included as a potential biomarker in human synovial fluid in the diagnosis and progression of OA patients by a clinical observational study in 2022 (NCT02664870).

### Other signaling factors

There are several other signaling cascades that have essential roles in the OA onset and development, such as notch signaling. Notch signaling was upregulated in mouse and human OA cartilage.^437^ Notch2 gain of function mutation in articular cartilage increased the severity of post-trauma OA by crosstalk with NF-κB, Wnt, and TGFβ signaling.^438,439^ Transient overexpression of NICD (NOTCH1 intracellular domain) led to enhanced synthesis of ECM and promoted the maintenance of articular cartilage, while constitutional overexpression of NICD resulted in early and progressive OA lesions in mice.^437^ Activation of Hes1, an essential mediator of Notch signaling, suppressed articular cartilage degradation and OA development by decreasing the Adamts5 and Mmp13 expression.^440^ The endoplasmic reticulum (ER) stress-triggered unfolded protein response (UPR) signaling has been identified as a contributing factor to OA pathology. Older OA patients developed ER stress in the early-stage OA when there was a higher synthesis of ECM proteins.^441^ How the UPR signaling mediated cell survival and the chronic ER stress-initiated apoptosis in cartilage and synovium contribute to the onset and development of OA needs to be investigated.

## Clinical therapy and clinical trials

So far there is no effective cure for OA. The OA treatment approaches are divided into physical modalities, pharmacologic treatments, and surgical treatments.^442^ Several new therapies have also recently been developed. In the early stages of OA, treatment focuses on reducing pain and joint stiffness. Subsequently, treatment mainly focuses on maintaining joint physical function.^443,444^ In summary, OA treatment aims to reduce the disease symptoms and slow its progression.

### Non-pharmacological treatment

#### Weight loss

Excessive body weight or obesity is a major risk factor for OA.^445^ Greater body weight adversely affects joint structure by adding additional load to the joints during daily activities and causing increases in the expression and production of enzymes that degrade the joints or increase joint inflammation.^446^ Weight loss is recommended for overweight or obese patients with low-extremity osteoarthritis.^447^

#### Exercise

According to the recommendations from the International Association for the Study of OA (OARSI), exercise is considered a core approach to the treatment of OA and is recommended for all patients.^448^ Exercise has been extensively studied as a treatment for OA.^448^ Uthman and colleagues found that exercise reduced painful movements and improved physical function in OA patients.^449^ The most common exercises used to treat OA include aquatic exercise,^450^ aerobic exercise,^451^ resistance exercise,^451^ multimodal exercise,^452^ and combination exercise.^452,453^

#### Assistive devices

OA patients often need assistive devices to compensate for decreased strength and impaired pain during exercise.^454^ Common devices include splints, braces, canes, functional shoes, and other training equipment.^455^ While there are some positive results from clinical studies, the need for assistive devices and their long-term safety remains in doubt.^456^

#### Physical therapy

Physiotherapy has significant therapeutic effects on OA, including therapeutic ultrasound, electrical stimulation, phototherapy, hydrotherapy, magnetotherapy, cryotherapy, and thermotherapy.^457–461^ Physical therapy provides significant relief of symptoms of OA, including pain, edema, and joint motion disturbances, and is suitable for emergency management in the acute phase.^460^ Instructing patients to use thermal agents has been recommended as a self-management strategy by the recent American College of Rheumatology Clinical Guidelines.^462^

#### Acupuncture

Acupuncture is a non-pharmacological treatment method in Chinese medicine.^463,464^ Acupuncture has analgesic and functional restorative effects in treating OA.^463^ The therapeutic effect of acupuncture may come from modulating inflammatory factors.^463,465^ However, there is evidence of uncertainty in the treatment of OA with acupuncture, particularly a significant difference between electro-acupuncture and hand acupuncture.^466^ In addition, non-pharmacological strategies, including health education, and lifestyle changes, such as diet, postural correction, and self-management, are important measures to prevent OA.^467,468^

### Pharmacologic treatment

Currently, no drugs can alter the progression of OA and prevent long-term disability.^469^ Current international guidelines recommend medications for the treatment of OA that revolve solely around reducing the burden of the disease (symptomatic effects) and altering the natural course of the disease by slowing or stopping the biological process of tissue damage.^469,470^ The following classes of drugs are currently used to treat OA of the knee: non-steroidal anti-inflammatory drugs (NSAIDs), glucocorticoids, opioids, symptomatic, chondroprotective agents, and anti-cytokines.^470–472^

Paracetamol (acetaminophen) is the first-line analgesic for clinical pain control of arthritis.^470^ The safety profile of paracetamol relative to other analgesics, such as non-steroidal anti-inflammatory drugs (NSAIDs), has led to its increased use, resulting in paracetamol becoming one of the most common drugs used in OA treatment.^470^ However, there is evidence that paracetamol is associated with an increased risk of gastrointestinal, cardiovascular, and renal disease, as well as mortality.^473^ NSAIDs are commonly used anti-inflammatory and analgesic drugs in the treatment of OA.^474,475^ NSAIDs, including ketorolac, and COX-2 inhibitors, such as diclofenac, ibuprofen, celecoxib, and rofecoxib,^474,476^ have finished phase 4 clinical trial (NCT01444365) in April 2012. Acetaminophen has been found to be effective in the treatment of OA, and acetaminophen is usually safe and may be better in situations where NSAIDs are contraindicated, has finished phase 4 clinical trial (NCT00635349) in April 2009.^477–479^ Glucocorticoids are another important class of drugs used in the OA treatment^480,481^ and finished the phase 4 clinical trial (NCT05291650) in Dec. 2022. When signs of inflammation appear, intra-articular injections of glucocorticoids can very quickly eliminate joint effusion and provide analgesia.^481,482^ Glucocorticoids include methylprednisolone acetate, trenbolone acetate (TA), betamethasone acetate (BA) and betamethasone sodium phosphate (BP), hexamethonium trenbolone (TH) and dexamethasone (DEX).^482–484^ They exhibit a complex biological activity, including anti-inflammatory and immunosuppressive effects that block the production of pro-inflammatory cytokines, leukocyte recruitment, and activation.^485,486^ Steroid injections should be used with caution in diabetic patients who are already hyperglycemic.^487,488^ Chondroprotective agents include montelukast, placebo, glucosamine, chondroitin sulfate, and hyaluronic acid.^489–491^ Montelukast and placebo entered the early phase I clinical trial (NCT04572256) in Feb. 2021. The mechanisms of action of individual factors have not been fully elucidated, and they range from inhibition of inflammation and injury receptor blockade to potential alterations in the viscoelastic properties of cartilage tissue.^490–492^ These agents slow the radiographic progression of knee osteoarthritis.^492–494^ Anti-cytokine treatments have also been considered.^495^ Many of the drugs used to treat OA are designed to counteract the pro-inflammatory, matrix-destroying effects of cytokines.^495^ Infliximab, a neutralizing antibody to TNF-α, finished the phase 4 clinical trial (NCT01144143) in June 2010. Further therapeutic approaches include the use of anti-TNF-α antibodies or the use of anti-inflammatory enzymes, such as IL-4, IL-10, IL-13, and TNF-β.^496–499^ Strontium ranelate inhibits subchondral bone resorption by modulating the activity of osteoprotegerin (OPG), RANK ligands, and MMPs produced by osteoblasts.^500,501^ Strontium may have a direct effect on cartilage and promotes the synthesis of proteoglycans and thus stimulates cartilage matrix formation in vitro.^500,502^ Intermittent injections of PTH (iPTH) reduce OA pain and slow the progression of OA by reducing subchondral bone degeneration and cartilage degeneration.^503^ In addition, iPTH reduces sensory innervation and PGE2 levels in subchondral bone and improves subchondral bone remodeling via PTH1R on Nestin^+^ MSC.^503^ Teriparatide entered the Phase 2 clinical trial (NCT03072147) in Mar. 2017. Nerve growth factor (NGF) is thought to modulate signals that control the expression of peripheral and central pain substances and enable neighboring nociceptive neurons to respond to stimuli.^478,504–506^ Tanezumab is a highly selective immunoglobulin G2 osteoarthritis13 antibody against NGF^507,508^ and finished the phase 3 clinical trial (NCT02709486) in Nov. 2018. Several studies have shown that tanezumab is more effective in improving knee pain, stiffness, and function in patients with moderate to severe knee osteoarthritis compared to placebo.^508–510^

### Biological treatment

Regenerative therapy has emerged as one of the most recent and rapidly evolving strategies for treating OA.^511,512^ Platelet-rich plasma extracted from blood samples provides important growth factors which may facilitate OA recovery.^511–513^ Platelet-rich plasma led to clinical improvements 12 months after injection and completed the phase 4 clinical trial (NCT02984228) in Aug. 2022.^511,513^ Mesenchymal stem cells (MSCs) related strategies for OA treatment have been extensively studied due to their ability to self-renew, immunomodulatory properties, and potential multilineage differentiation ability (especially toward chondrocytes).^514–517^ Intra-articular injections of autologous MSCs, usually derived from bone marrow or adipose tissue, finished the phase 4 clinical trial (NCT04675359) in Jul. 2021.^514,517,518^ Several clinical studies using MSCs-based therapy for regeneration of cartilage in knee OA have reported positive results.^519,520^ Kim and co-workers injected adipose-derived MSCs into 49 patients and measured treatment outcomes by the IKDC (International Knee Documentation Committee) scores, Tegner activity scores, and overall patient satisfaction with the procedure.^521^ Results showed that the mean scores of IKDC and Tegner activity were significantly improved both preoperatively and postoperatively, while they noted that patient age and lesion size were important factors affecting clinical outcomes.^521^ In 2019, Chahal et al. published a non-randomized, open-label, dose-escalating phase I/II clinical study,^522^ which included twelve 40–65-year-old patients who received different doses of bone marrow-derived MSCs. The follow-up trials from baseline to 12 months indicate that patients showed significant improvements in KOOS and WOMAC hardness scores, quality of life, and symptoms 12 months after BM-MSC treatment.^522^ In addition, patients treated with higher doses showed better outcomes than those treated with lower doses.^522^ Furthermore, gene therapy is a new therapeutic approach tested in clinical for OA treatment in recent years. TGF-β1 gene therapy using retroviruses was approved for allogeneic chondrocytes in vitro.^523^ Kim and coworkers reported the clinical safety and efficacy of the intra-articular injection of tissue gene-c (TG-C) in OA patients. TG-C is a cell-based gene therapy that uses un-transduced and transduced chondrocytes that are retroviral transduction of overexpressing TGF-β1.^524^ They concluded that TG-C was associated with statistically significant improvements in function and pain in patients with knee OA and started the phase III clinical trial (NCT03291470) in Oct. 2021.

### Surgical treatment

As OA continues to worsen, surgery becomes necessary when basic treatment and medications are ineffective.^525^ The goal of surgery for OA patients is to reduce pain, decrease disability and improve quality of life.^526^ With the help of modern technology, patients can usually bear weight on the affected limb immediately after surgery.^527^

#### Knee arthroscopy

Knee arthroscopy is the most used clinical procedure with minimal invasion to treat OA.^528^ It can be used to detect injuries of joints, repair injured soft tissues, such as ligaments and tendons, and bones, and remove inflamed and damaged tissue. For articular cartilage, surgical strategies include scraping (debris removal and chondroplasty), repair (microfracture and perforation), or restoration (autologous chondrocyte graft, autologous osteochondral graft, and allogeneic osteochondral graft).^529^

#### Artificial joint replacement

Arthroplasty is currently an effective clinical treatment for advanced knee OA. It effectively eliminates pain, corrects joint deformity, and improves knee function.^530^ However, clinical studies have found that some patients still do not recover satisfactorily after surgery and cannot fully straighten the knee.^531^ Cartilage injury, ligament injury, postoperative infection, and postoperative deep vein thrombosis in the lower extremity are all factors that influence the outcome of surgical treatment of knee OA, and early postoperative prevention of complications is crucial.^531,532^

## Conclusions and perspectives

OA is a tremendously complex synovial whole-joint disorder, and how OA is initiated and developed remains poorly understood. Research on the cellular and molecular mechanisms of OA is still in the beginning phase. We have summarized from the current knowledge changes of key molecules in the essential signaling pathways in the articular cartilage, synovial membrane, subchondral bone, and synovial fluids of OA patients and animal models (Table 3), as well as the potential therapeutic reagents that have been reported (Table 4). Wnt, TNF, TGFβ/BMP, FGF pathway receptors, FA proteins, and other factors that are located on the chondrocyte membrane sense and transduce biochemical and mechanical signals. Activated signaling pathways and regulators, such as AMPK, mTOR, FGF, BMP, HIFs, and NF-κB, via crosstalk and feedback mechanisms in a complicated network, regulate the expression of key downstream factors, such as Runx2, MMP13, ADAMTS4/5, Prg4, and other factors, in articular chondrocytes and synovium. The initiation factors of OA are various, including excessive mechanical loading, inflammatory factors, aging and etc., which lead to the different primary effects of early-stage OA with unique molecular signaling signatures. The destruction and erosion of the articular cartilage, the synovial hyperplasia and inflammation, the abnormal angiogenesis of the synovial joint, the subchondral bone disturbance, and the instability of the ligaments and tendons could all contribute together or respectfully to the onset and progression of the disease. Scientists and clinic doctors are still debating, and there is still no conclusion on which one happens earlier than the others during the initiation of OA. No matter which pathological factor is the first dominant over others, or they contribute equally to the progression of OA; it has been well accepted that interference at the early stage of OA to prevent its progression will be a more efficient and effective strategy for better outcomes than focusing on the medical treatments and joint replacement surgery at the late and end-stage of the disease. Therefore, targeting critical signaling pathways and key molecules that are significantly changed at the early stage of the disease to control crosstalk between molecular pathways and the interaction of different compartments of synovial joint is critical for future research.

**Table 3:** Therapeutic reagents and their strategies and target genes have been reported for each OA pathogenic signaling pathway

Signaling pathway	Key protein	Articular cartilage	Synovial membrane	Subchondral bone	Joint cavity (synovial fluid)	References
Wnt	Wnt	Increated in DMM-induced OA in mice; Increased in human OA cartilage	Increased in human OA cartilage	unknown	Increated in DMM-induced OA in mice; Increased in human OA cartilage	PMID: 22682243;27147711
	WISP-1	Increased in human OA cartilage	Increased in human OA cartilage	Increased in human OA cartilage	Increased in human OA cartilage	PMID: 19180479
	β-catenin	Increased in human OA cartilage	Increased in human OA cartilage	Increased in human OA cartilage	Increased in human OA cartilage	PMID: 19180479;15866164
	Dickkopf-1	unknown	unknown	unknown	Decreased in human OA cartilage	PMID: 28678406
AMPK pathway	AMPKα	Decreased in DMM-induced OA mouse model and Human knee OA chondrocytes.	Decreased in acute gouty arthritis (AGA) rats.	unknown	unknown	PMID: 25940958;30585453
	AMPKβ	unknown	unknown	unknown	unknown	n.a
	AMPKγ	unknown	unknown	unknown	unknown	n.a
NF-κB	RelA/p65	P65 protein is released, phosphorylated, and translocated from the cytoplasm to the nucleus in DMM-induced OA in mice; p65 translocates to the nucleus in human OA cartilage	P65 protein is released, phosphorylated, and translocated from the cytoplasm to the nucleus in DMM-induced OA in mice; p66 translocates to the nucleus in human OA cartilage	P65 protein is released, phosphorylated, and translocated from the cytoplasm to the nucleus in DMM-induced OA in mice; p67 translocates to the nucleus in human OA cartilage	P65 protein is released, phosphorylated, and translocated from the cytoplasm to the nucleus in DMM-induced OA in mice; p68 translocates to the nucleus in human OA cartilage	PMID: 31842396
	RelB	RelB protein is released, phosphorylated, and translocated from the cytoplasm to the nucleus in DMM-induced OA in mice; RelB translocates to the nucleus in human OA cartilage	RelB protein is released, phosphorylated, and translocated from the cytoplasm to the nucleus in DMM-induced OA in mice; RelB translocates to the nucleus in human OA cartilage	RelB protein is released, phosphorylated, and translocated from the cytoplasm to the nucleus in DMM-induced OA in mice; RelB translocates to the nucleus in human OA cartilage	RelB protein is released, phosphorylated, and translocated from the cytoplasm to the nucleus in DMM-induced OA in mice; RelB translocates to the nucleus in human OA cartilage	PMID: 31842396
	c-Rel	c-Rel protein is released, phosphorylated, and translocated from the cytoplasm to the nucleus in DMM-induced OA in mice; c-Rel translocate to the nucleus in human OA cartilage	c-Rel protein is released, phosphorylated, and translocated from the cytoplasm to the nucleus in DMM-induced OA in mice; c-Rel translocate to the nucleus in human OA cartilage	c-Rel protein is released, phosphorylated, and translocated from the cytoplasm to the nucleus in DMM-induced OA in mice; c-Rel translocate to the nucleus in human OA cartilage	c-Rel protein is released, phosphorylated, and translocated from the cytoplasm to the nucleus in DMM-induced OA in mice; c-Rel translocate to the nucleus in human OA cartilage	PMID: 31842396
	NF-κB1/p105p50	The precursor protein p105 opposed to degradation and release of active p50; p50 protein is released and translocated from the cytoplasm to the nucleus in DMM-induced OA in mice; p50 translocate to the nucleus in human OA cartilage	The precursor protein p105 opposed to degradation and release of active p50; p50 protein is released and translocated from the cytoplasm to the nucleus in DMM-induced OA in mice; p51 translocates to the nucleus in human OA cartilage	The precursor protein p105 opposed to degradation and release of active p50; p50 protein is released and translocated from the cytoplasm to the nucleus in DMM-induced OA in mice; p52 translocates to the nucleus in human OA cartilage	The precursor protein p105 opposed to degradation and release of active p50; p50 protein is released and translocated from the cytoplasm to the nucleus in DMM-induced OA in mice; p53 translocates to the nucleus in human OA cartilage	PMID: 31842396
	NF-κB2/p100p52	The precursor protein p100 opposed to degradation and release of active p52; p50 protein is released and translocated from the cytoplasm to the nucleus in DMM-induced OA in mice; p52 translocates to the nucleus in human OA cartilage	The precursor protein p100 opposed to degradation and release of active p52; p50 protein is released and translocated from the cytoplasm to the nucleus in DMM-induced OA in mice; p53 translocates to the nucleus in human OA cartilage	The precursor protein p100 opposed to degradation and release of active p52; p50 protein is released and translocated from the cytoplasm to the nucleus in DMM-induced OA in mice p54 translocate to the nucleus in human OA cartilage	The precursor protein p100 opposed to degradation and release of active p52; p50 protein is released and translocated from the cytoplasm to the nucleus in DMM-induced OA in mice p55 translocate to the nucleus in human OA cartilage	PMID: 31842396
HIF pathway	HIF1a	Decreased in DMM-induced OA in mice; Increased in human OA cartilage.	Upregulated in MIA-induced KOA in rats.	Upregulated in osteoblasts in human OA subchondral bone.	Increased in human OA cartilage.	PMID: 27122313; 28123469
	HIF2a	Increased in STR/ort and DMM mouse OA model. Increased in human OA cartilage.	Unknown	Unknown	Unknown	PMID: 28123469; 24999110
	HIF3a	Unknown	Unknown	Unknown	Unknown	
mTOR	mTOR	Increased in DMM-induced OA in mice; Increased in human OA cartilage.	Unknown	No change	Unknown	PMID:24651621
Focal adhesion pathway	Kindlin-2	Highly expressed in healthy articular cartilage; Decreased in aged and OA cartilage	Unknown	Unknown	Unknown	https://www.nature.com/articles/s43587-021-00165-w
	Talin	Decreased in human OA cartilage	Unknown	Unknown	Unknown	
	Vinculin	Decreased in human OA cartilage	Unknown	Unknown	Unknown	
	Integrin αVβ3	Unknown	Elevated in synovial membrane in DMM mouse model.	Unknown	Unknown	PMID:31534047
	Focal adhesion kinase	Unknown	Unknown	Elevated in subchondral bone in ACLT rat model.	Unknown	PMID:32324278
BMP pathway	BMP-2	Increased in human OA cartilage; Down-regulated in DMM-induced OA in rats	Unknown	Increased in human OA cartilage; Increased in ACLT-induced OA in rats	Decreased in patients with OA	PMID: 35216203; 32290085; 29429984
	BMP-4	Increased in human OA cartilage; downregulated in DMM-induced OA in rats	Unknown	Increased in human OA cartilage	Unknown	PMID: 35216203
	BMP-6	Upregulated in DMM-induced OA in rats	Unknown	Unknown	Unknown	PMID: 34680516
	BMP-7	Decreased in human OA cartilage; upregulated in DMM-induced OA in rats	Unknown	Unknown	Unknown	PMID: 29980690; 26010756
	GDF-5	Down-regulated in DMM-induced OA in rats	Unknown	Unknown	Unknown	PMID: 26010756

*FRZB* frizzled-related protein, *IκB* inhibitors of NF-κB, *IKK* IκB kinases, *HIF* hypoxia-induced factor, *PHD* HIF prolyl hydroxylases, *AAV* adeno-associated virus

**Table 4:** The expression and changes of FGF signaling in human and animal synovial joint and OA

Signaling pathway	Therapeutic strategy	Target gene	Therapeutic reagent	References
Wnt	Inhibition of Wnt protein	Wnt	SM04690	PMID: 28978991
	Inhibit Wnt signaling in an indirect way by upregulating FRZB expression	FRZB	Verapamil	PMID: 24658359
AMPK pathway	Upregulated phosphorylated AMPK (AMPK activator)	AMPKα1	Metformin;	PMID:32156705; 25940958
		AMPK-mTOR signaling pathway	Vitamin D	PMID:31815524
		AMPK/PGC-1α pathway.	Puerarin	PMID:29961054
		AMPK-Nrf2 Signaling	Luteolin	PMID:35154568
	Promoted SIRT1 expression.	SIRT1	Safflower yellow (SY)	PMID:32871523
			17β-E2	PMID:33613450
NF-κB	Induce expression of IκB, causing increased cytosolic retention of NF-κB	I-κB	Glucocorticoids, NF-κB ODN, NF-κB morpholinos.	PMID:10760263
	Inhibit IKK activity significantly, preventing IκB phosphorylation	IKK	Non-steroidal anti-inflammatory drugs (NSAIDs), such as aspirin, salycilate, ibuprofen, and sulindac	PMID:11641233
	Preventing IκBα degradation	20 S proteasome complex	Bortezomib, Cyclosporin A, sc-514, lactacystin.	PMID:15135296
	Diminution of levels	p65, p50	siRNA, AAV	PMID:11160335
	Inhibit Nuclear translocation of NF-κB subunits p65, p50	p65, p50	FK-506, BMS-205820, I-κB super repressor, Tat-srIκBα.	PMID:8112299
	Inhibit the NF-κB DNA-binding activity	p65, p50	Glucocorticoids, NF-κB ODN, NF-κB morpholinos.	PMID:10760263
HIFs pathway	Inhibition of PHD protein (PHD inhibitor)	PHD2	TM6008, TM6089	PMID: 28682438
		FK506-binding Protein	FKBP38	PMID: 28682438
		PHD1/2/3	DFO, DMOG	PMID: 28682438
	Inhibition of HIF2a protein (HIF2 inhibitor)	The silent information regulator 2 type 1 (SIRT1)	Resveratrol	PMID: 25737402
		HIF2a	YC-1	PMID: 25737402
mTOR	Inhibition of mTOR	mTOR	Rapamycin	PMID:22084394
			The hydromethanolic extract of Butea monosperma (BME)	PMID: 28987943
Focal adhesion pathway	Intra-articular injection of Kindlin-2-expressing AAV	*Fermt2*	AAV	https://www.nature.com/articles/s43587-021-00165-w
	Inhibition of FAK (FAK inhibitor)	*PTK2*	PF-0455487;	PMID:32558123
			Y15	PMID:32324278
BMP pathway	Inhibition of BMP2 protein (BMP2 inhibitor)	BMP2	Noggin	PMID: 32290085
	Inhibition of BMP4 protein	Rspondin2	Mianserin	PMID: 30808932
			MiR-181a	PMID: 34778258
	Activation of BMP7 pathway	Smad1/5	BMP7-derived peptide	PMID: 33850953
	Activation of BMPRIa pathway	BMPRIa	CK2.1	PMID: 28420447

*KOA* knee osteoarthritis, *DMM* destabilization of the medial meniscus, *MIA* monosodium iodoacetate

The multiple layers of complexity of the pathogenesis of OA and limited knowledge of the pathogenetic molecular signaling pathways and specific mechanisms have made the therapeutic pharmacological targeting of OA extremely difficult. At this point, in the future, a comprehensive understanding of alterations in distinct signaling pathways and expression of key factors in the superficial, middle and deep zones of articular cartilage and synovial membrane at different stages of OA triggered by different factors should be investigated in great detail. The whole picture of functions and regulations of each pathological signaling and key factors in various early-stage OA conditions could help us to develop more specific resolutions to halt or reverse the OA disease.
